# A systems approach for optimizing implementation to impact: meeting report and proceedings of the 2019 *In the Trenches: Implementation to Impact International Summit *

**DOI:** 10.1186/s12919-020-00189-x

**Published:** 2020-07-24

**Authors:** Stephen R. Hanney, Pavel V. Ovseiko, Kathryn E. R. Graham, Heidi Chorzempa, Maxi Miciak

**Affiliations:** 1 Health Economics Research Group, Brunel University London, Uxbridge, Middlesex, UB8 3PH UK; 2 Radcliffe Department of Medicine, University of Oxford, John Radcliffe Hospital, Oxford, OX3 9DU UK; 3 Alberta Innovates, 1500, 10104-103 Avenue NW, Edmonton, AB T5J 0H8 Canada; 4 Faculty of Rehabilitation Medicine, University of Alberta, 8205-114 St. NW, Edmonton, AB T6G 2G4 Canada

**Keywords:** Research impact assessment, Implementation science, Innovation, Research, Systems approach, Engagement, Alberta Innovates

## Abstract

**Background:**

The *In the Trenches* series of cutting-edge knowledge sharing events on impact for front-line experts and practitioners provides an engagement platform for diverse stakeholders across government, research funding organizations, industry, and academia to share emerging knowledge and practical experiences. The second event of the series *In the Trenches: Implementation to Impact International Summit* was held in Banff, Alberta, Canada, on June 7–8, 2019. The overarching vision for the Summit was to create an engagement platform for addressing key challenges and finding practical solutions to move from implementation (i.e. putting findings into effect) to impact (i.e. creating benefits to society and the economy).

**Processes and proceedings:**

The Summit used diverse approaches to facilitate active engagement and knowledge sharing between 80 delegates across sectors and jurisdictions. Summit sessions mostly consisted of short talks and moderated panels grouped into eight thematic sessions. Each presentation included a summary of Key Messages, along with a summary of the Actionable Insights which concluded each session. The presentations and discussions are analysed, synthesized and described in this proceedings paper using a systems approach. This demonstrates how the Summit focused on each of the necessary functions (and associated components) that should be undertaken, and combined, for effective research and innovation: stewardship and governance, securing finance, creating capacity, and producing and using research. The approach also identifies relevant challenges.

**Conclusions:**

There is increased interest globally in the benefits that can accrue from adopting a systems approach to research and innovation. Various organizations in Canada and internationally have made considerable progress on *Implementation to Impact*, often as a result of well-planned initiatives. The Summit highlights the value of 1) collaboration between researchers and potential users, and 2) the adoption by funders of approaches involving an increasing range of responsibilities and activities. The Summit website (https://inthetrenchessummit.com/) will be periodically updated with new resources and information about future *In the Trenches* events.

## Background

Research and innovation (R&I) is the engine of sustainable social progress and economic growth. R&I is created across disparate sectors and communities of practice, therefore, experts and practitioners implementing research and assessing its impact at the front-line require an engagement platform. The *In the Trenches* series of cutting-edge knowledge sharing events on impact for front-line experts and practitioners provides an engagement platform for diverse stakeholders across government, research funding organizations, industry, and academia to share emerging knowledge and practical experiences.

Building on the success of the International School for Research Impact Assessment (ISRIA) [1], the series was launched with the inaugural *In the Trenches: Research Translation for Health Impact International Symposium* held at the University of Oxford, United Kingdom, on November 16, 2018 [2–4]. Supported by the National Institute for Health Research (NIHR) Oxford Biomedical Research Centre [5], it had a focus on assessing and optimizing the translational value of health R&I using co-creation approaches, whereby researchers and practitioners engage with multiple stakeholders to devise products and services that increase their value for everyone.

The second event of the series *In the Trenches: Implementation to Impact International Summit* was held in Banff, Alberta, Canada, on June 7–8, 2019. The Summit was organized by Alberta Innovates (AI) – Alberta’s research and innovation corporation – and co-hosted with the University of Oxford. As part of its mandate, AI provides advice and connections to stimulate and grow R&I in Alberta with a view to contributing to a diversified economy, cleaner and sustainable environment and healthier communities [6]. The Summit is an example of AI’s convener role bringing together communities of practice that have the potential to contribute to solving the grand challenges in these priority areas and address the valley of death in implementing and scaling innovations for achieving impact. The challenge of “bridging the valleys of death” with a greater understanding of the “time lags” between critical points across the research and innovation pipeline is a topic of growing interest by those interested in maximizing the rate of returns from R&I investments [7]. To address societal problems, a system approach is needed, one that aligns strategic priorities with stakeholder interests and values. To do this, AI recognizes the need to be responsive and engage stakeholders across the R&I lifecycle from the generation of solutions to involving end-users in adopting and scaling innovations to achieve intended impacts.

The Summit had a focus on scale and spread of R&I in the local Albertan, national Canadian, and relevant international contexts. The overarching vision for the Summit was to create an engagement platform to address key challenges and find practical solutions to implementing and scaling innovations for achieving impact. The aspiration is to accelerate and optimize the impact of R&I by having implementation experts share their knowledge and practices with impact experts and vice versa. The aims of the Summit were:

- Advance the science of implementation and impact by engaging in interdisciplinary dialogue.
- Share leading approaches and frameworks for implementation and impact, with a particular focus on scale and spread of R&I in local and international contexts.
- Engage the implementation and impact communities in a lively dialogue regarding common challenges and ways of working together to move implementation to impact in complex environments.
- Facilitate networking opportunities between impact and implementation communities, across sectors and jurisdictions.

Looking to the future and in the words of Laura Kilcrease (CEO, Alberta Innovates) *“what we need to do here in Canada, and Alberta specifically is expand our views beyond our borders… we have to get our innovation into a product, get the product into a tangible market, and then make the linkages and connections to scale the firm globally, wherever that maybe around the world”.*

## Processes and proceedings

### Overview

The Summit brought together 80 leading R&I professionals, including program managers, evaluators, knowledge translation practitioners, research impact assessment and implementation scientists, entrepreneurs, policy makers, and patient representatives. Summit delegates represented organizations in healthcare (39%), R&I funders (18%), academia (17%), not-for-profits, industry and the business community (12%), government and media (4% each respectively) and others (6%). Among the delegates in attendance, 57% worked within the health sector. While a 74% majority of delegates were from Alberta and other Canadian provinces (8%), 19% of delegates were international – from Australia, Austria, Denmark, Netherlands, Spain, the United Kingdom (UK), and the United States of America (USA).

### Program development

The Summit Program (see Additional File 1) was developed by the International Organizing Committee and co-directed by Drs. Kathryn Graham (Alberta Innovates) and Pavel Ovseiko (University of Oxford). In response to feedback on the initial idea for the Summit conceived by Graham, and newly emerging ideas from stakeholders and presenters, extensive iterations of the event schedule and line-up of presenters were made. The presenters determined the format and refined the content of every session via teleconference calls and email communication. The planning, organization, stakeholder engagement, promotion and communication, and post-Summit evaluation were undertaken by the AI team with input from the Alberta Innovates Advisory Committee. An additional file lists the Members of the International Organizing Committee and the Alberta Innovates Advisory Committee (see Additional file 2).

### Promotion and communication

The Summit was promoted to potential delegates in Canada and internationally via email, social media, and the AI website. The following groups were specifically engaged to promote local, national, and international participation and expertise in implementation and impact:

#### International R&I funding organizations

The Agency for Health Quality and Assessment of Catalonia (AQuAS), the NIHR in England, the Netherlands Organization for Health Research and Development (ZonMw), and the Commonwealth Scientific and Industrial Research Organization (CSIRO) in Australia were considered key international stakeholders given their work advancing research impact and implementation.

#### Canadian Institutes of Health Research (CIHR)

CIHR is Canada’s national funding body for health research and is committed to assessing the impact of its investments.

#### National Alliance of Provincial Health Research Organizations (NAPHRO)

Members of this Canadian organization were considered for their expertise in health impact and impact assessment, R&I system knowledge, and extensive network of stakeholders.

#### Alberta Health Services (AHS)

Alberta delivers health services through AHS, the province’s single, integrated health system. Participation of AHS implementation practitioners and scholars would enhance dialogue and promote collaborations to address local challenges.

#### Alberta Strategy for Patient-Oriented Research SUPPORT Unit (AbSPORU)

Patient-oriented research is a potential mechanism for advancing implementation and impact of R&I. Engaging AbSPORU and its network would provide insight into challenges and potential solutions to scaling health research innovations for impact at local and national levels.

#### Government of Alberta ministries

The Ministry of Economic Development and Trade (now Ministry of Economic Development, Trade, and Tourism) and the Ministry of Health were invited to participate due to their strategic investments in provincial R&I and governance across sectors (e.g. Health, Energy).

#### Government of Canada ministries

The Ministry of Science (now Ministry of Innovation, Science and Industry) and the Ministry of Small Business and Export Promotion (now Ministry of Small Business, Export Promotion and International Trade) were engaged given their role in R&I policy and governance in industry at the federal level.

#### Academic institutions

Alberta universities were considered a key stakeholder given their role within the local R&I ecosystem and in advancing implementation science.

#### Cross-sector industries

In addition to the health sector, stakeholders from energy and environment (e.g. Emissions Reduction Alberta), Agriculture (e.g. Ministry of Agriculture and Forestry in Alberta), and technology (e.g. InnoTech) were engaged to provide broad perspectives on implementation and impact.

#### Business and innovation community

The Summit was promoted as part of a wider program of INVENTURE$ connect events occurring concurrently in Alberta. These events were organised by AI to bring venture capitalists, angel investors, startups, entrepreneurs, service providers and thought leaders together to discover and share the latest in innovation, research, capital access and deal-making. This extended the potential reach to national and international delegates with expertise in the scale of innovation in business.

Seven months prior to the Summit, a save-the-date flyer was circulated to the specific groups. Members of the International Organizing Committee promoted the Summit via personal engagement and presentations at relevant meetings and conferences. For 3 months leading up to the event, information about the Summit and the subsequent updates were posted on Twitter using a dedicated hashtag #implementation2impact.

### Summit format and peer-to-peer engagement

The Summit had a 1.5-day format commonly used for in-person scientific meetings [8]. Summit sessions mostly consisted of short presentations and moderated panels grouped into eight themes (see Additional File 1). Each presentation included a summary of Key Messages, and every session concluded with a summary of Actionable Insights. The opening and closing keynote addresses by Pavel Ovseiko and Stephen Hanney helped to frame the event themes in context and tie concepts discussed at the Summit to theory and or frameworks used in implementation science and impact assessment. The keynote speakers also contributed to the Summit’s synthesis of Key Messages and associated Actionable Insights.

An overarching goal of the Summit was to facilitate meaningful and authentic engagement among delegates (presenters included) to promote networking, formal and informal learning, and collaboration. To achieve this aim, the engagement strategy used during the Summit focused on creating a collegial and relaxed environment to encourage open and ‘out of the box’ exchange using various and frequent touch points both inside and outside of formal program sessions. While many of the features of the Summit may be found at other conferences, the extent of the range of engagement approaches used is noteworthy:

#### Seating

The Summit venue was arranged “cabaret” style, with seating comprised of round tables with an open end facing the stage. This arrangement encouraged delegates to interact and exchange opinions during Summit sessions.

#### Delegate packs

Included in the delegate’s event package was a contact list of those who consented to share their information, and a Speaker Summary sheet outlining the biographies and experience of those speaking at the event: delegate feedback suggested these resources were particularly valuable in facilitating connections both during the event and after. Among the 80 delegates in attendance, 31 (39%) participated as Keynote speakers, session presenters and panellists. An updated contact list was distributed post-Summit to help delegates maintain newly established connections and encourage future contacts and collaborations.

#### Social connection activities

Each day opened with an icebreaker activity to foster connections between delegates and closed with an evening social event to provide further opportunity for networking. For example, the first day opened with an activity that had delegates find a fellow delegate they had not previously met, then tell each other where they were from and the most interesting thing about that place.

#### Social media

Twitter was used both for traditional online dissemination and engagement as well as to facilitate personal connections through a contest. The “My New Best Friend Forever (BFF)” contest had delegates take selfies with a delegate(s) they just met at the Summit and post to Twitter with the hashtags #implementation2impact and #newBFF.

#### Dialogue with presenters

Ensuring contact between speakers and other delegates was an explicit intention of the organizers and was addressed not only with general networking sessions, but also with, “Dialogues with Presenters” opportunities. Here session speakers made themselves available for further engagement among those interested: these Dialogue moments provided interested delegates with opportunities for critical discussion and reflection with presenters. In addition to providing opportunity for dialogue, this activity was designed to cultivate a collegial and collaborative environment that reinforced peer-to-peer engagement (i.e. presenters were considered peers).

#### World Café sessions

To achieve the Summit’s aim of engaging implementation and impact communities to address complex translational challenges, organizers felt it was essential to provide a small group activity to facilitate active participation of all delegates. Therefore, in addition to the presentations and panel discussions, the concurrent small group sessions in the World Café provided an opportunity to gather delegate input about practical actions that could be taken to advance implementation and impact efforts and barriers to progress in these areas. Concurrent sessions enabled frank dialogue and reinforced opportunities to forge connections between delegates.

#### Exhibition table

The Exhibition Table was a designated area that reinforced engagement and learning through the display of knowledge and promotional products made available to delegates. Delegates could broaden their knowledge of the work being done by the organizations and groups represented at the Summit by visiting the exhibition stand which included research articles and print copies of select reports and guidelines mentioned at the event, as well as other items including badges and other promotional and engagement materials.

### Summit website

A Summit website (https://inthetrenchessummit.com/) was launched 2 months prior to the event. The website initially contained information about the Summit date and location, schedule, speakers, and how to register. A Resources page was added to the website: there seminal pre-reading items identified by confirmed speakers were posted for those interested in learning more about the Summit’s topics and themes in advance of the event. After the Summit, the website’s Resource page was updated to facilitate further post-event engagement and learning. The Resources page contains 25 Summit presentations, Open Access articles, and other relevant materials selected by the speakers. The website will be periodically updated with new resources and information about future *In the Trenches* events.

### Summit evaluation

An online post-event evaluation of the Summit was conducted. The purpose of the evaluation was to assess delegate experience and achievement of objectives and was used gather information to inform plans for the *In the Trenches* series. In total, 23 of 77 delegates invited to participate responded to the survey (30% response rate).

**Fig. 1:**
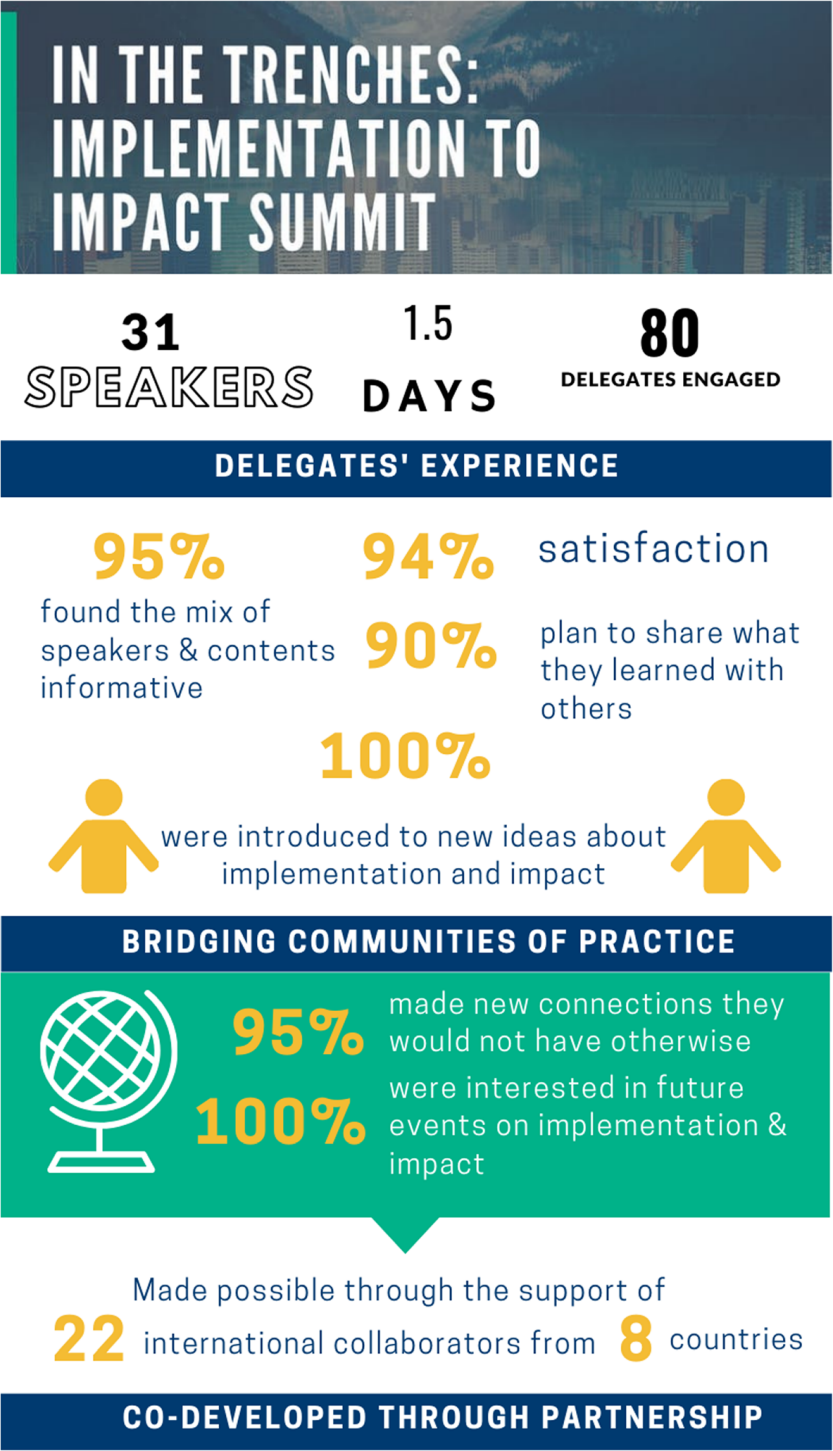
In The Trenches Summit Infographic of Delegate Evaluation

Overall, nearly all respondents (94%) were satisfied with the Summit and most (79%) would recommend *In The Trenches* events to others. The achievement of objectives was also rated favorably, with scores for the seven objectives ranging between 86 and 100% (Fig. 1).

Respondents reported that opportunities to meet new people and learn about the application of implementation and impact considerations in scale and spread projects and initiatives from different perspectives was the most useful aspect of the event. Nevertheless, several respondents identified the need for more practical information on implementing some of the ideas presented. At approximately 7-weeks following the event, 48% of those surveyed had subsequently engaged or followed up with another delegate for the primary purpose of ongoing knowledge exchange and sharing. Some reported additional reasons for engagement, as this respondent’s quote illustrates: “*Following up on ideas and shared experiences; discussing possibilities for future collaboration; making friends.”*

Analytics from the Summit’s social media channels – the event website and twitter hashtag – were also examined to understand the reach and engagement achieved through these mechanisms. In total, the website received 3178 page views from 1069 new or unique visitors; the most common sources of website traffic were through visitors directed to the site (74%), referral from others (16%), social media (4%) and organic searches (3%). Additionally, the event hashtag (#Implmentation2Impact) was tweeted and retweeted more than 200 times with those messages receiving 343 likes.

### Systems approach

In his closing remarks summarising the Summit, Hanney noted how the presentations from some organisations clearly suggested they were adopting a systems approach as they described their various actions to boost progress in aspects of the R&I journey. Additionally, the first session of the World Café (Session 6A) was explicitly described as involving a dialogue about *“a ‘systems approach’ to innovation achieving impact.”* Furthermore, the focus throughout the Summit on engagement and interactions between different stakeholders, and the increasing roles for research funders, facilitates – in some ways almost demands – the adoption of a systems approach in the analysis of the Summit’s proceedings. Therefore, post-Summit discussions within the team of authors of this paper, and some others involved with the Summit (see Acknowledgements), led to agreement on the potential value of incorporating a systems approach into the current proceedings because it:

- provides a structure for systematically analysing what was said at the Summit about *Implementation to Impact* in ways that do justice to the richness of the discussions;
- highlights and draws on how some funders are already making progress in applying aspects of a systems-type approach, which is also helping to generate, and in turn being strengthened by, an impact culture and enhanced sustainability; 
- facilitates a demonstration of the implementation and impact benefits associated with embedding a health R&I system into the healthcare system;
- illustrates potential ways of addressing the challenges of *Implementation to Impact* and tensions that were identified at the Summit; and
- identifies ways in which progress could be made across the board in promoting *Implementation to Impact.* Here, ways can be identified also to boost key themes that were common across various presentations.

Therefore, the proceedings will be organized around points a) – e) above and presented in three sections. These three sections are briefly introduced here and will then be presented in full in Proceedings Sections 1-3.

In the first section, *analysing the presented material according to the eight components of a (health) research system,* the components of a health research system framework will be used to organize an interpretive account and analysis of the diverse Summit presentations. The most appropriate starting point is deemed to be the health research systems framework developed for the World Health Organization (WHO) [9]. This health research systems framework has four functions, each with one or more components. The functions cover the full range of activities necessary if adopting a systems approach going from stewardship and governance, through securing finance and building capacity, to producing and using research. Informed by this, but amending it for our purposes, the eight operational components to be used here are listed below:

1. Define and Articulate Vision for a (Health) R&I System
2. Identify Appropriate (Health) R&I Priorities
3. Set and Monitor Ethical Standards for (Health) R&I and Research Partnerships
4. Monitor and Evaluate the (Health) R&I System
5. Secure Funding for (Health) Research and R&I System Building
6. Build, Strengthen and Sustain the Human and Physical Capacity to Conduct, Absorb and Utilize (Health) Research
7. Produce Scientifically Valid (Health) Research Outputs
8. Communicate and Promote Research to Inform (Health) Policy, Practices and Public Opinion and to Develop Tools (e.g. Drugs and Devices) to Improve Health, Society and the Economy.

Aspects of all the components were mentioned at the Summit, even though in some cases they did not form the basis of a specific session. Furthermore, while this framework was explicitly developed for a national system, and for health research, the presentations by contributors from other fields, including from organizations that funded research in multiple fields, can also be described using the above list of components. More recent analysis for the WHO suggests a crucial factor for a successful research system is ensuring as comprehensive and coherent coverage of the various components as possible [10]. A study conducted to inform public involvement in health research in Ontario, Canada, recently used the WHO health research system framework to analyse and learn lessons from systems where such public involvement was particularly well developed. The two case studies therefore focused on Alberta and England [11].

In the above list of components the original WHO framework has been specifically adapted to help analyse/promote *Implementation to Impact*. For example, the financing component in this context also relates to securing resources for conducting the diverse activities necessary to organize a systems approach to facilitating *Implementation to Impact*. Usually, each national research system has its own diverse pattern of funders, therefore, the framework should be seen more as a way of organizing thinking and items to consider, rather than as providing a precise blueprint or template for construction of an R&I system. All the presentations from a single session are described together in Additional file 3, but some presentations – or specific points from them – will be discussed separately from the rest of their session where that assists coverage of the components and facilitates comprehensive coverage of the scope of the Summit. Furthermore, various presentations, or the discussions they informed, will be drawn upon in the account of more than one component.

In Section Two, *Discussion of emerging themes and challenges,* the various presentations and discussions (outlined in Additional file 3) will be drawn upon to analyse each of points b), c) and d) above, as outlined here in the following three bullet points:

- In relation to the development of a systems approach, many of the presentations illustrated that certain research systems, and/or major funders, are already making progress along these lines. These developments will be analysed, by considering how adoption of a comprehensive and coherent systems approach means the various specific elements will be mutually reinforcing and help to generate, and in turn be strengthened by, an impact culture and enhanced sustainability. These organizations include three major funders of health research mentioned above from Spain, England and the Netherlands respectively: AQuAS, NIHR, and ZonMw. Additionally, while AI covers various fields, much of the focus at the Summit was on its role in the health field. CSIRO in Australia also funds research in many fields.
- Some presentations, especially from AI, illustrated how research systems are being embedded into the relevant healthcare system. Evidence about the feasibility and benefits of this approach will be analysed.
- Despite accounts of progress in scaling and spreading R&I results more broadly, some Summit presentations and discussions provided examples of challenges and tensions facing *Implementation to Impact,* and the role of a systems approach in facilitating ways to address them. Such challenges and tensions include, for example:
  - how to reduce the time often taken to achieve implementation and impact and yet allow time to build up the appropriate structures, capacity and learning;
  - how to address the inevitable resistance to change from both producers and potential users of research;
  - the many and genuine difficulties facing attempts to increase coproduction in research;
  - the need to avoid the previously reported high rate of research waste [12] and to provide value; and
  - the importance of ensuring the robustness of the research before attempting to achieve impact by implementing the findings, which also has implications for the balance between use of local and global research.

Section three, *Lessons and further work,* will describe ideas proposed at the Summit for progressing *Implementation to Impact,* through encouraging a systems approach to incorporate the various components. This section also explores the possibility of developing a new framework to build upon and advance this work. Additionally, some practical guides relevant to points made in the Summit will be listed, even in some cases when they were not specifically referenced in the presentations. Finally, the possibility of taking these ideas forward in future events will be explored.

## Section one: analysing the presented material according to the eight components of a (health) research system

### Component one: define and articulate vision for a (health) R&I system

In the context of *Implementation to Impact,* it is important that the research system, or funder, should not only articulate a vision, but also that the vision should emphasize the importance of the research making a societal impact. Early examples of text that today might be seen as vision and/or mission statements were not necessarily called that at the time. Peter Riddles (CSIRO) (Session 2A) highlighted that in Australia the original *Science and Industry Research Act 1949* establishing the CSIRO had referred to the utilisation of research. According to the Act, CSIRO’s functions were as follows [13]:

(a) to carry out scientific research for any of the following purposes:

(i) assisting Australian industry;

(ii) furthering the interests of the Australian community;

(iii) contributing to the achievement of Australian national objectives or the performance of the national and international responsibilities of the Commonwealth;

(iv) any other purpose determined by the Minister;

(b) to encourage or facilitate the application or utilization of the results of such research;

(ba) to encourage or facilitate the application or utilisation of the results of any other scientific research;

In the Key Messages from his presentation, Riddles described various ways in which business innovation had evolved in Australia, and that agencies such as CSIRO had a role in this (Session 2Aii – see in Additional file 3).

In the World Café discussion session that specifically considered *“a ‘systems’ approach to innovation achieving impact”* it was suggested that a *“shared vision”* was one of the innovation enablers (6Ai). The importance of nurturing key values within a research system was highlighted in contributions such as that from Adam Kamenetzky (Actionable Insights, Session 5E). He claimed, *“equality, diversity and inclusion are fundamental aspects that ought to be considered at all levels of the research and innovation ‘ecosystem’*”. Vision and mission statements provide opportunities for the values of the R&I system to be promoted.

### Component two: identify appropriate (health) R&I priorities

When the concern is *Implementation to Impact,* it is even more important than usual that the priority setting is appropriate to the needs of the intended users. The involvement of relevant stakeholders was a major theme throughout the Summit.

Some of the presentations/sessions focused on the engagement of a wide range of stakeholders in identifying topics, or setting the agenda, for the research that would meet the needs of the relevant system. Others, especially session 4, focused specifically on the importance of engaging the patients and public.

Jean Miller, a patient representative in Alberta and member of Patient and Community Engagement Research (PaCER), emphasized the importance of meaningful participation of patients in R&I in order to achieve implementation and impact (Session 4A). Her top Key Message was: “*First find out what’s important to patients and study that”’* (4Ai). In discussion, she also described how experienced patient representatives should be part of the team providing training to new patient representatives in order to enable them to fully participate.

Lauren Gerlach from AcademyHealth in the USA described various ways of facilitating and promoting patient engagement throughout the research lifecycle (Session 4C). In her presentation, she also illustrated a practical tool developed by the Patient-Centered Outcomes Research Institute (PCORI) in the USA that provides information on the engagement of patients and other stakeholders in research. Their *Engagement in Health Research Literature Explorer* database [14] allows the body of relevant literature to be searched by items such as types of stakeholder, and phases of research engagement going from identifying research questions to dissemination and uptake of results.

Various important practical points about how to support patient engagement were also made in the discussion in session 4. Examples include offering support for patients to engage in research by providing, for example, lunch and childcare support. Mechanisms to ensure patient involvement were also discussed, for example, by making it a condition of funding. (Guidelines covering much of this are also available on the website of INVOLVE, the part of the NIHR in England that promotes public and patient engagement in health research [15]).

In one of his Actionable Insights from session 4, Tim Murphy identified patients as the primary audience for the results and efforts related to R&I in health, and that “*Partnership improves the quality and relevance of researchers’ work and it also empowers the patient partners”* (4D). Therefore, those building R&I systems should *“Design and implement research and innovation programs and initiatives with this central partnership concept in mind”* (4D)*.* A similar concept was identified in themes from the World Café discussion, *Addressing Sustainability in Real-World Applications* (Session 6B): “*Sustainability research needs the patient lens as a design-level lens”* (6Bii).

While Component Two of the (health) research system very much focuses on research priority-setting, in session 4, and other instances, Summit speakers also described how stakeholder engagement should continue after the topic identification at meaningful points along the R&I continuum. Therefore, the important aspects of stakeholder engagement are applicable to other components of R&I systems, as illustrated in the Key Messages raised by organizations including the Ontario Brain Institute (OBI) (Session 4B). Some Summit presentations focused on the role of stakeholder engagement in developing impact assessment strategies – these are mostly considered in Component Four on monitoring and evaluation (M&E).

The overlaps are particularly complex in the comprehensive Health Research and Innovation Assessment System (SARIS) being developed by AQuAS in Catalonia and described by Núria Radó-Trilla in session 3B. In the context of ongoing engagement with stakeholders to develop the system of assessment, SARIS also encourages stakeholder engagement in research “*to promote impact”*. In her presentation, Radó-Trilla gave the example of a programme of nursing research where the engagement was with patients, in particular, to understand research needs.

Similarly, in describing the comprehensive approach towards implementation and impact taken by CSIRO, Tom Keenan declared “*Researchers must engage effectively with end users and other key stakeholders at appropriate stages across the impact pathway to ensure the desired impact is realized”* (Session 3Cii). This approach was amplified in Keenan’s presentation where he explained that at the CSIRO, they believe the whole organization is responsible for impact, and the relationships built with end users/stakeholders should be long-term, if possible, and built on trust.

**Fig. 2:**
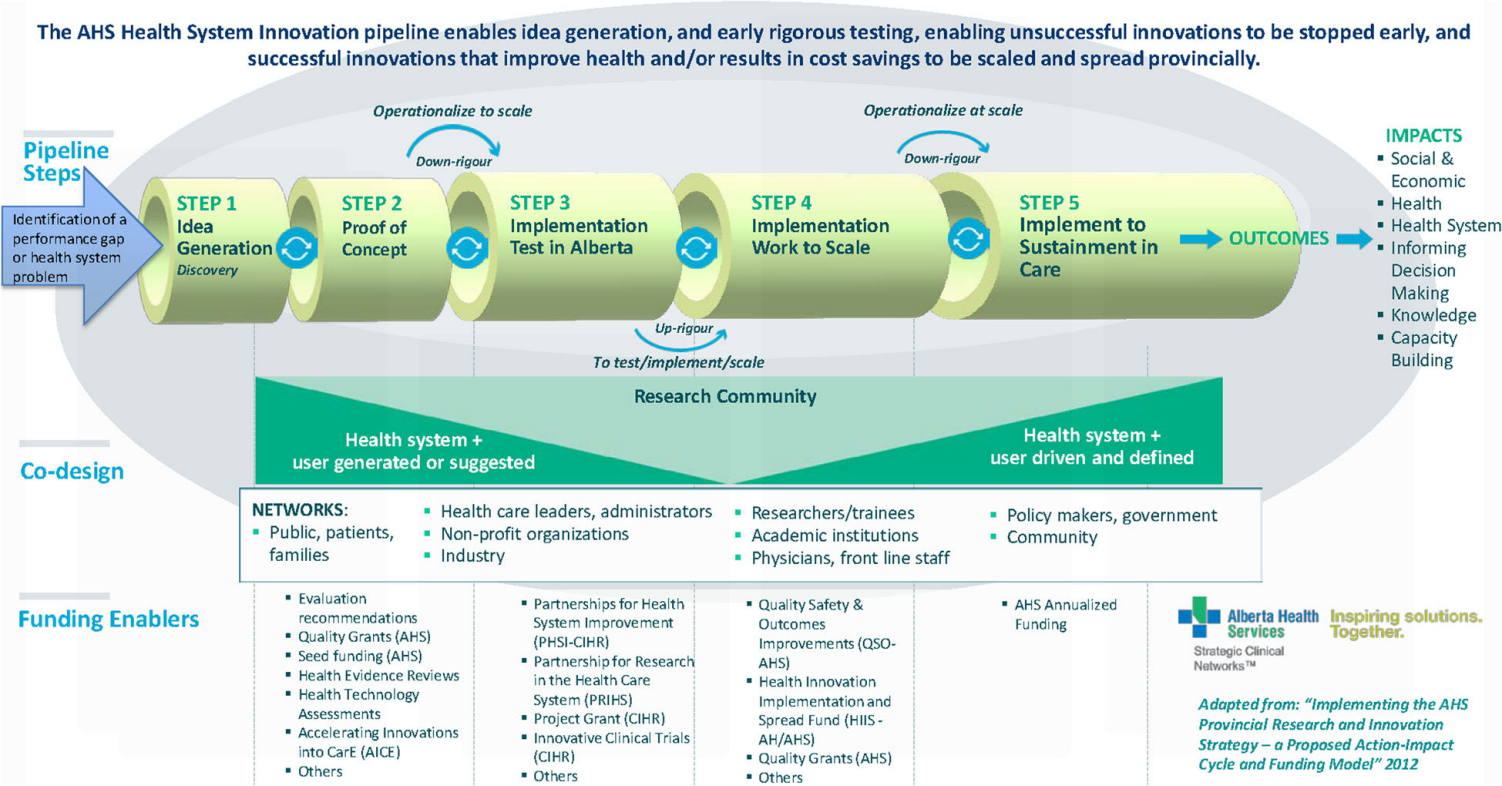
The Alberta Health Services (AHS) innovation pipeline. Adapted from AHS Research Strategy, 2012 [16]. Reproduced with permission

In session 7, a team from Alberta described *The Alberta Health Services (AHS) Innovation Pipeline* (Fig. 2). Here, the co-prioritisation process involves a comprehensive network of stakeholders who identify gaps in the care provided. This goes further than many other approaches, because the co-design informs a search for existing R&I that might address the needs, as well as topics for new research. Tim Murphy (Session 7A) described the importance of moving towards open models of innovation with wide participation to bridge the *Implementation to Impact* gap.

The AHS has created various Strategic Clinical Networks (SCNs). In session 7B, Nancy Fraser from the Critical Care SCN described how in Alberta’s Partnership for Research and Innovation in the Health System (PRIHS) all members are part of the network *“coming together to solve problems and advance care….a bi-directional dialogue that allows the health system to identity gaps and feed them into the research community to help solve”* (7Bi). Building on this, Jeffrey Crelinsten, Moderator of session 7, recommended the following action to Summit delegates and the R&I community more broadly: *“actively engage patients, service providers and payers with researchers in the identification of gaps and problems and in the development and testing of solutions”* (7F).

As set out by Murphy in his introductory welcome to the Summit, AI believes more is achieved by working together across sectors covering many fields. Therefore, in addition to the work in the health field, other aspects of AI’s approach were described, as were links with a parallel organization, Emissions Reduction Alberta (ERA). In session 2, Elizabeth Shirt described the importance of collaboration for ERA in enabling it to address challenges (2Cii), and explained it also takes a portfolio approach – working on some innovations that can be implemented rapidly to meet a need, and recognising that others will take longer reinforcing the importance of stakeholder relations for successful implementation and subsequent impact sustainability.

### Component three: set and monitor ethical standards for (health) R&I and research partnerships

The issue of research ethics is important across scientific fields, and often particularly so for health research. The International School on Research Impact Assessment (ISRIA) statement was made available at the Summit and provides a 10-point guideline for an effective process of research impact assessment [1]. Point eight covers ethics specifically in relation to research impact assessment by saying: *“Anticipate and address ethical issues and conflicts of interest”.*

Also, in relation to *Implementation to Impact,* during the discussion following the presentations in session 4, Jean Miller referred to an especially pertinent document on the ethics of engaging patients in research [17]. This Draft Ethics Guidance was developed by a CIHR Working Group made up of patients, researchers and ethicists, and is intended as an educational resource primarily for patients and health researchers, as well as research institutions and funders of research. The Guidance focuses on ethical concerns that need to be addressed to maintain trust in research partnerships across the research lifecycle. A public consultation on the Draft Ethics Guidance ran from November 26, 2018 to February 25, 2019 and the feedback received during the consultation was reported as being used to inform the final version of the document [17].

### Component four: monitor and evaluate the (health) R&I system

The monitoring and evaluation (M&E) component in the original Pang et al. (2003) framework [9] was located here in the sequence rather than at the end. This is now of crucial importance for this report because it demonstrates how those working in R&I systems can be encouraged to realize that impact will be a major element of the M&E system. This, in turn, provides both incentives and justifications for focusing on activities likely to maximize impacts. This was one of the Key Messages in the presentations from Hanney (1Biii) and others on impact frameworks.

Hanney described in session 1B that the creation of the Payback Framework in the 1990s [18] involved adding the assessment of wider societal impacts to the traditional assessment of academic excellence that primarily focused on knowledge production and research capacity building. The Payback Framework was informed by earlier UK research that pioneered concepts around collaborative agenda setting between potential users of research and the researchers [19] and this led to Hanney’s Key Message that: “*Impact assessment frameworks such as the Payback Framework are informed by models of collaboration and implementation”* (1Bi)*.* The Payback Framework provides a combination of the various categories of academic and wider benefits or impacts, with a model of how to organize their assessment. In practical terms, this framework is then used to inform the various methods that can be used to assess the impact, including documentary analysis, surveys, interviews and case studies – see Hanney et al. (2013) for examples [20].

The original Pang et al. (2003) framework for Health Research Systems [9] incorporated the assessment of wider impacts from the Payback Framework, which also helped to inform the Canadian Academy of Health Sciences (CAHS) framework developed by a committee chaired by Alberta’s Cy Frank. Graham explained how the CAHS framework: *“built on the Payback Framework’s academic and societal impacts; provides a common set of tools, including indicators****;****identifies pathways to impact across the five payback categories…”* (1Biv). An account of how the CAHS framework has been applied is available in the proceedings of a forum held in Alberta in 2015 for delegates from across Canada [21].

In session 7A, Murphy argued that in order to meet various requirements along the Innovation Pipeline (Fig.2*), it would be necessary to replace the traditional “fund and forget”* funding model with a new type of *“value hunter/value optimizer”* R&I funder. The focus would be on value generation complemented by an assessment approach that includes “*indicators and metrics which give line of sight to the intended impact – and we can measure it”* (7Aii). In session 9a, Alan O’Connor and Kathryn Graham shared the impact assessment framework used to assess the socioeconomic return on the investment in the PRIHS program described above by Fraser as part of the Innovation Pipeline. They noted that, “*Having a shared vision of impact sustained the partnership over the years”* (9Ai). Their presentation focused on three components of the impact assessment framework. First, the ‘stage gate approach’ to support decision making across the R&I life cycle as reflected in the pipeline. Second, the impact framework to monitor and evaluate which includes an economic model and performance measures for assessing scale up and spread of R&Is for social returns including improved patient health. And third, communicating results, which involves a scorecard and “*impact narratives”* (9Aiii).

As noted above, some R&I funders are not only building impact assessment approaches that encourage a focus on impact throughout research and implementation processes, but also engage stakeholders in developing the impact assessment approach. In her presentation, Radó-Trilla from AQuAS explained how the organization’s SARIS approach incorporated the *“Need to move from an excellent-centered policy to a mixed strategy of excellence and impact”* (Session 3B). The SARIS approach is closely linked to the ISRIA Statement that was authored by an international team led from AQuAS [1]. The impact narratives developed under SARIS to provide accountability can then be analysed to provide lessons from a specific funding stream for future R&I investment opportunities. There is a challenge to engage parts of the evaluation community to adopt the new approaches to research assessment.

Summarizing lessons learned from other research funders that would be relevant for the NIHR’s approach to impact [22], Kamenetzky concluded that “*engaging researcher communities & wider stakeholders should be at the core of impact pathway planning, and subsequent research impact assessment”* (1Cii). Furthermore, he argued, it was important to recognize the benefits that could come from assessing impacts, including *“greater mutual understanding between funders and researchers, improved communications, and better evidence of value”* (1Civ).

The focus of session 9 was engaging stakeholders in assessing and improving impact in order to sustain it. In session 9B, Maxi Miciak from AI described a Canadian collaborative approach to develop an assessment framework for health services and policy decision-making. The aim was that the inclusion of many organizations in developing a shared framework would increase the likelihood of it being adopted [23]. Miciak and Graham, in collaboration with CIHR and its Institute of Health Services and Policy Research (IHSPR), played a central role in coordinating the collective work of Alliance members in developing the assessment framework; in her presentation Miciak highlighted the importance of the collaborative approach as taken here. Her first Key Message was, *“Co-development and implementation of impact assessment frameworks and plans fuel stability through confidence, resonance, and relevance”* (9Bi). She also observed that, *“Different groups are at different stages of readiness in terms of need, capacity, and ability, so providing options for engagement with impact assessment will promote sustainable scale and spread of impact assessment”* (9Bii).

Similarly, in session 9D Carrie Hough and Adam Kamenetzky explained how a new Global Health Research programme from England’s NIHR is aiming to design a monitoring, evaluation and learning approach to maximise sustainable impact. The NIHR is seeking to *“collaboratively identify pathways to impact; establish mechanisms for testing whether these pathways do or do not work; identify emerging lessons to feed into portfolio development; and, embed monitoring evaluation and learning considerations throughout the portfolio”* (9Di). Hough and Kamenetzky discussed adopting a portfolio level Theory of Change to inform the next steps and emphasized the importance of collaborations to develop the framework. In his moderator’s Actionable Insights for session 9, the UK’s Ovseiko stated in his first Insight: *“Canadians in general and Albertans in particular have developed not only world-class, but in many respects, world-leading expertise in implementation to impact. It builds on learnings from the world’s best practice and is attuned to their unique context and needs”* (9E)*.*

### Component five: secure funding for research and R&I system building

In the context of this report, the key resource issue is not just the money to conduct research, but also, in particular, the resources required by (health) R&I systems, and funders, to undertake the various steps to encourage implementation and impact and, accordingly, the appropriate assessment of impact resulting from investments in R&I. Kamenetzky highlighted the importance of resource allocation for successful and sustainable implementation and impact in his Key Messages to funders: “*consider upfront the resources required for embedded, relevant and methodical approaches to impact & its assessment”* (1 Ci).

This message was amplified by Ovseiko, who argued *“As more information on the use of the established frameworks and approaches is becoming available and the development and implementation of new ones is continuing, it is becoming even more important for funders to commit sufficient resources to the assessment of research impact as well as to the implementation of research into practice”* (1D). After the speakers had described the work their respective organizations were undertaking to promote implementation and impact assessment, some discussions focused on practical questions about the resources available to conduct such activities. Generally, the presenters said their teams and/or budgets were small, but while they could do with more resources, they were all making progress. And, as emphasized by Reesa John from AI, it is important for R&I funders who adopt an approach that could be described as being ‘comprehensive and coherent’ to identify and show the value of their approach in order to justify their investments.

### Component six: build, strengthen and sustain the human and physical capacity to conduct, absorb and utilize (health) research

Pang et al. 2003 included building the human and physical capacity to absorb and utilize health research as part of the capacity building function in their original framework for a health research system. These aspects of capacity building were discussed at the Summit, along with promoting gender equity in R&I and the key contribution that health service data can make to the production, use and spread of relevant research findings and impact assessment.

The importance of developing sufficient capacity was highlighted in Ovseiko’s opening presentation in session 1: *“Implementation to impact is more of an art than a science: there are many useful tools, but implementation success ultimately depends on the capabilities of local staff to operate in complex systems*” (1Ai). Session 5 was devoted to various aspects of building the capacity for *Implementation to Impact,* mostly but not entirely from the perspective of activities underway in the health field.

In session 5A, Meghan McMahon described the role of the Health System Impact Fellowship program funded by the Canadian Institutes of Health Research – Institute of Health Services and Policy Research (CIHR-IHSPR) [24]. This scheme is attempting to respond to the changes in PhD graduate employment trends, and the emergence of learning health systems. It is preparing PhD trainees and post-doc fellows for success and impact in a range of roles within and beyond academia. According to McMahon, emerging lessons from assessment of the capacity development program *“suggest five ‘program ingredients’ are helping prepare fellows for stronger and more diverse careers: experiential learning within a health system organization, protected time for academic research, co-mentorship from health system and academic leaders, professional development training allowance, and participation in a national cohort”* (5Aii).

On a similar theme, Alex Clark (Session 5B) described how the increasing attention on impact in post-secondary institutions meant there was a need to create *“cultures that support and incentivize impact”* (5Bi). He argued that impact skills need not be separate streams of professional development but claimed *“Policy and experts recommend that training and support be provided for impact across the career trajectory, including: to graduate students, post-doctoral fellows and researchers. Individuals enhancing their skill should engage in professional networks”* (5Biii).

Diverse aspects of R&I capacity building were also featured elsewhere in Summit discussions. For example, Hough and Kamenetzky described the Global Health Research programme from England’s NIHR where stakeholders are exploring ways to establish *“proportionate monitoring and evaluation systems that support further capacity building for implementation and impact activities across the portfolio”* (9Diii). In session 7 on the Innovation Pipeline, Gabrielle Zimmermann from the Alberta Strategy for Patient Oriented Research SUPPORT Unit (AbSPORU) Knowledge Translation (KT) Platform described how one of the key initiatives of the platform *“is to advance implementation science in Alberta. We do this in part by providing advice and assistance with the practical application of implementation science, but we are also exploring the development of an implementation science lab for long-term impact”* (7Di). She explained how the Platform operates at the middle to end of the Innovation Pipeline (Fig. 2), and how Implementation Science labs *“can help build relationships and lay the foundation for robust processes within the healthcare system”* (7Diii) that support successful scale and spread of innovations for impact.

Capacity building for R&I broadly defined includes provision of infrastructure support. Laura Hillier described various ways in which infrastructure funding from the Canada Foundation for Innovation (CFI) supports the scale and spread of innovation in any field. It can be used strategically to build or support expertise and leadership in selected research areas, for example CFI supports the Ocean Tracking Network. This is a global research, technology, data-management and conservation platform headquartered at Dalhousie University in Halifax, Nova Scotia. The network supports research documenting the movements and survival of more than 130 species of aquatic animals carrying various types of electronic tags. Hillier’s first Key Message added to the major Summit theme of collaboration by stating: “*It is through strong collaboration linkages that new ideas become innovations and yield benefits to Canadians – research infrastructure can stimulate and support these collaborations*” (2Bi).

**Fig. 3:**
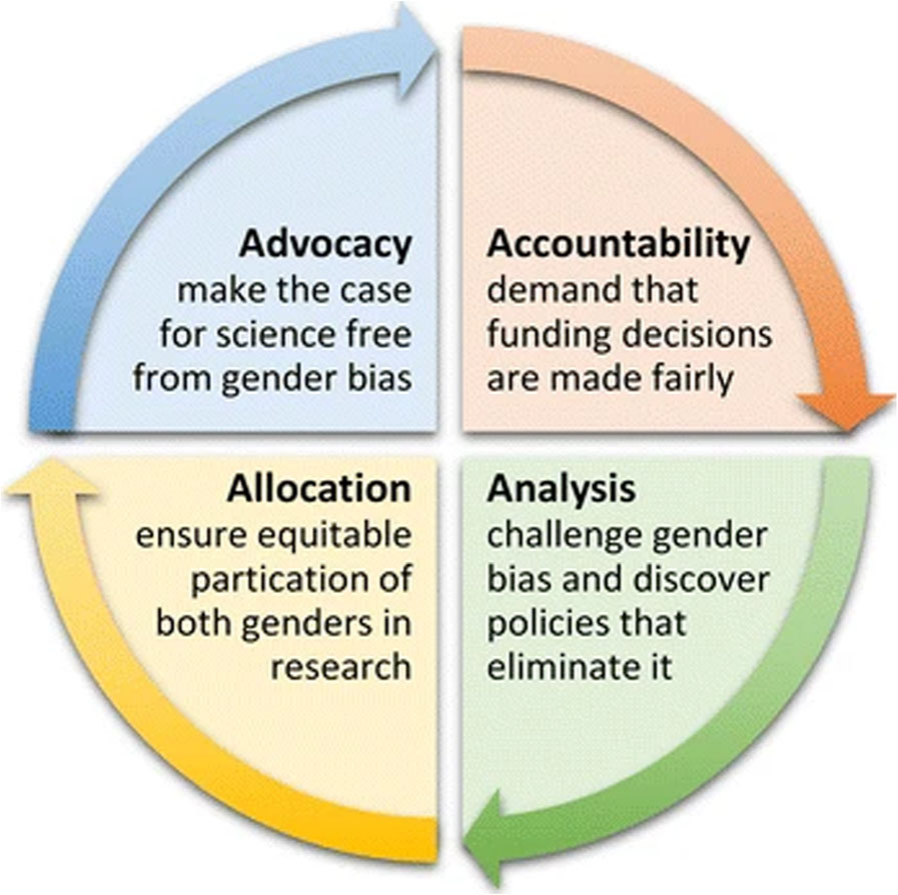
The Four “As” of research impact assessment with regard to gender equity. Source: Ovseiko et al. (2016) [25]

Two presentations focused on the specific issues around how promoting greater gender equity should enhance research impact. In session 5C, Ovseiko set out in considerable detail the evidence to support the idea that increasing gender equity should: “*Increase reproducibility of basic research”* (5 Ci)*, “Enhance translation of clinical research”* (5Cii) and “*Maximize the potential of the scientific workforce”* (5Ciii). He also made recommendations for research funders, institutions and evaluators by drawing on the paper he co-authored with a large team of international experts on how to include and strengthen analysis of gender equity in research impact assessment [25]. That team developed the approach, or tool, called the four ‘As’ of research impact assessment with regard to gender equity (Fig. 3).

Building on this call for action, Eduard Güell from AQuAS argued that the research impact agenda would continue to be incomplete unless the gender perspective is introduced, and female talent optimized. He claimed that there was a link between women’s participation in health science and attention to gender related and sex-related factors in disease-specific research. He described an ongoing project on the gender perspectives in biomedical science in Catalonia organized around the 4 ‘As’ of research impact assessment developed by Ovseiko et al. (2016) [25] and described above. He claimed: “*our pilot in mental health helps us to see our own bias and the importance of including a gender perspective when assessing research impact”* (5Div).

In commenting on the work from AQuAS, Kamenetzky suggested: *“Equity of impact cannot be achieved without efforts to understand and take action to address inherent and systemic biases: the work of AQuAS showed that while ‘looking into the mirror’ (to explore issues of gender bias in R&I) could be challenging, there was clear value in holding organisations to account, if collectively we are to move from being blind to these biases”* (5E).

Finally, one panel at the Summit focused entirely on data strategies and analytics that address data use. Increasing access to relevant data can be an important part of improving the capacity to conduct, scale-up and assess R&I. Dale Sanders’s presentation entitled, *Data-driven insights for accelerating scale-up and spread,* described many problems with the accuracy and access to US healthcare data. He called for an acknowledgement: *“that every piece of data that is collected as a consequence of healthcare delivery, is an artefact of data that ultimately belongs to the patient – not healthcare systems, researchers, governments, or clinicians – and should be shared and utilized to its fullest value for the benefit of patients”* (8Aiv). Alba Velasco Trujillo from SIRIS Academics described the approach developed at SIRIS to advance analytics for R&I assessment: *Semantic technologies and ontology-based data access for research impact monitoring and evaluation.* SIRIS recognizes the importance of data in evidence-based policy but identified that often there are challenges such as a lack of integration of existing data. New approaches, such as open data, are providing ways of addressing the challenges. For example, SIRIS has applied the new approaches to research innovation and impact assessment: *“Semantic Technologies solutions offer a disruptive toolkit to support the integration of data that are currently dispersed and highly heterogeneous and ensure they are accessed in an integrated, unified and semantically consistent way”* (8Biii).

The data landscape in Canada includes several high functioning cross-jurisdictional data sources and platforms. Nevertheless, according to Rick Glazier from CIHR-IHSPR, the situation is “*mainly characterized by fragmentation and lack of comparability between and within sectors”* (8 Ci). Recently, there has been increasing recognition of the importance of healthcare data to research, though Glazier cautions *“Much work is needed to regulate, govern, set standards, remove access barriers, and harmonize digital health, community and social services data in Canada”* (8Ciii). Commenting on the work required to improve the data landscape, Dale Sanders advised delegates that: *“Every strategy for becoming “data driven” should dedicate a significant portion of the strategy and project plan to building trustful relationships with those affected by the data… both the providers of data and the consumers of the data”* (8D).

### Component seven: produce scientifically valid (health) research outputs

Various presenters, including Hanney and Graham (1B), in relation to the Payback Framework and CAHS Framework respectively, as well as Keenan (3C) and Radó-Trilla (3A) emphasized the continuing importance of traditional research outputs (e.g. scientific publications) when an additional evaluation focus is added to assess the wider impacts of R&I.

The importance of the production of scientifically valid outputs was central to the presentation by Wendy Reijmerink from ZonMw in the Netherlands (Session 9C). While she also described ZonMw’s approach to encouraging impact (see next component), she emphasized the importance of first focusing on the quality of the research production along with the societal relevance: *“Don’t talk about impact when the underlying evidence is not robust in terms of relevance and quality”* (9 Ci). These comments were made in the context of a stream of work in *The Lancet* related to the claim originally made by Chalmers and Glasziou (2009) [12] that estimated the annual avoidable waste in research production and reporting was up to 85%. A further series of papers in 2014 [26–28] discusses the five stages of waste in research, including: the relevance of the questions; the design, conduct and analysis; and non-publication. In her presentation (9C), Reijmerink then highlighted the Consensus Statement from The Ensuring Value in Research (EViR) Funders’ Collaboration and Development Forum [29]:

- *“we set justifiable research priorities;*
- *we require robust research design, conduct and analysis;*
- *we seek to ensure that research regulation and management are proportionate to risks;*
- *we seek to ensure that complete information on research methods and findings from studies is accessible and usable.”*

She drew on them when describing the ZonMw framework for fostering responsible research practices that covers the various elements in the funders’ Consensus Statement. These include setting justifiable research priorities that should ensure that the questions are relevant to the users of research [30]. To this, the concept of productive interactions is most helpful [31].

As noted in Component Two, many of the presentations on the engagement of patients and other stakeholders emphasized that such engagement helps improve the relevance of the research priorities. But it goes further, and various presentations also argued that later stages such as the conduct of research and production of valid outputs were also improved by stakeholder engagement. To facilitate this, Gerlach at AcademyHealth advises organizations contemplating patient engagement to consider “*How might traditional definitions of evidence (including what’s considered rigorous) need to change to better incorporate patient narratives, insights from local communities, and other important data sources?”* (4 Ci). Likewise, Murphy stipulated “*A re-engineering of the current evidence production process is required and will assist and support more effective production, dissemination, and implementation of research”* (4D).

### Component eight: communicate and promote research to inform (health) policy, practices and public opinion and to develop tools (drugs and devices) to improve health, society and the economy

In the opening keynote presentation Ovseiko set the scene for the complexities involved in *Implementation to Impact* and referred to it as being *“more of an art than a science*” (1Ai). In the second World Café session, *Addressing Sustainability in Real-world Applications,* contributors identified a series of reported barriers to sustaining efforts to implement R&I results into real-world contexts. In addition to difficulties in monitoring and evaluating progress, the session moderators from Alberta, Kelly Mrklas and Rachel Flynn, reported: *“contextual barriers such as: resourcing (costs drive the decision making); implementation and learning climate (e.g. the complexity of health systems; silos within and across the healthcare system that prevent individuals from learning from one another around issues of common and mutual concern); and implementation readiness and culture (i.e. moving past the traditional “pilot project” mentality and shifting to longer lines of sight involving scale, spread, sustainability and impact)”* (6Biv).

Many of the above issues relate to the organization within which the implementation solution and its sustainability is supposed to be taking place. Mrklas and Flynn additionally argued that “*The people part of change*” (6Biii) was a key consideration, and this point was also emphasized by Dale Sanders, who proposed that *“Every investment strategy in innovation should include an evaluation of its likelihood of adoption based on the fundamentals of human behavior”* (2D).

Various presentations at the Summit focused on aspects of encouraging implementation, and what could be achieved by developments in research systems as well as systems in which research results are implemented. There were descriptions of holistic, or system-wide, attempts to increase implementation, scale and impact, and accounts of the importance of culture in achieving implementation and impact. Such points were brought together in one of the World Café sessions: “*To influence the system to perform better at ‘capturing the benefit’ the government needs to: achieve a balance of encouraging, enabling and enforcing, e.g. supporting culture changes and providing resources, but also holding people to account and being willing to shut down things that aren’t delivering”* (6Aiii).

Two presentations used the metaphor of a house. While they did so in different ways, both were appropriate for highlighting the need for the type of ‘comprehensive and coherent’ approach described earlier as a key feature of effective R&I systems that will translate research findings and achieve the range of impacts. Session 3 focused on the role of stakeholder engagement in culture change. In the first presentation, Reesa John described AI’s Integrated End to End Impact Management System, in a presentation entitled, *Creating an organizational culture to move implementation to impact.* She emphasized that researchers need to remember their “*project exists within a system that is made up of a community of people*” (3Ai), it is culture that *“has the power to unite that community to ignite and inspire impact”* (3Aii), and that *“Integrating a Culture plan up front as part of your Impact plan is critical to success”* (3Aiii) in achieving broader impact. In describing *“the house that impact built”* she suggested that culture could provide the frame for the house and within that there would be many elements which organizations can construct to achieve impact. These include an integrated end-to-end impact management system, mechanisms to support system coordination and partnerships across stakeholders and sectors (e.g. collaborative funding programs, adoptions of new requirements and opportunities for training in integrated knowledge synthesis and stakeholder engagement), coordinated investment portfolios in focused areas, and an impact assessment strategy including evaluation.

In their presentation on NIHR’s approach to evaluating the impact of the health research funded by the Global Health Research programme, Hough and Kamenetzky also described the importance of culture. They explained how “*Activities to create an ‘impact culture’ for this work by engaging across the various points of the health research system have initially focussed on participatory activities with research applicants…and peer review committee members”* (9Dii). Just as John had done, they stressed the importance of working in a community, but in this case illustrated the point by showing that the community of a village was stronger than an individual house.

The importance of culture in *Implementation to Impact* also featured in Keenan’s presentation. He described CSIRO’s impact framework which covers the full spectrum of activities from initial engagement (as noted for Component Three) and inputs, which can be controlled and planned, through to those such as outcomes and impact on which the funder has less control. Nevertheless, “*Effective planning for impact is crucial for the ultimate delivery of that impact through the uptake & adoption of research ‘babies’”* (3 Ci). He also described how CSIRO had been working on developing long-term relations with users for 10 years, and that *“Embedding impact culture in research organisations takes time – and requires both ‘top down’ & ‘bottom up’ approaches”* (3Ciii). In the opening session, one of Kamenetzky’s observations for the NIHR from the approach of other funders was: ***“Take time:****orienting research to societal and economic domains of impact is a long-term process of strategic change for funders and researchers”* (1Ciii).

The continuing thread across Summit speakers that stakeholder engagement in R&I systems facilitates implementation and impact is also seen in the Ontario Brain Institute’s approach to stakeholder engagement. First, *“Co-developing tools and resources for patients empowers them with knowledge, levels the perceived power dynamic, and supports their ability to become active partners in their own treatment and care”* (4Bii). But it goes further than this because *“Developing partnerships with community-based organizations extends the reach of these tools and helps scale and spread knowledge from the bottom up”* (4Biii).

Reijmerink’s presentation illustrates how ZonMw (described previously) is fostering responsible research to maximize sustainable impact in terms of observable use of valuable evidence, promoting for example the adoption of real-world quality/innovation cycles in its programming processes. Her third Key Message proclaimed: *“Smart funding agencies steer on accumulation of small wins, aimed at better health and healthcare for all”* (9Ciii).

While building the structures to enhance *Implementation to Impact* can take a long time, one of the aims of the activities stressed in various presentations is to speed up as well as increase the processes that facilitate implementation. This aim featured in Elizabeth Shirt’s presentation on the work of ERA in developing clean technology in Alberta (Session 2C). She introduced Summit delegates to a series of Technology Readiness Levels (TRLs) and the three elements that needed to be brought together to make progress along those levels. She called the three: Toolset; Team-set; and Mindset. Her emphasis on the importance of collaboration, as described in Component Two, was part of the team-set element. She described how the toolset included: *“tools to identify and accelerate technologies (and companies) along the TRL scale towards commercialization”* (3 Ci).

The Summit session *Accelerating Scale and Spread for Sustainable Impact – A Local Approach* (Session 7) emphasized the importance of attempting to reduce the time taken to realize wider benefits from R&I*.* In that session, Murphy added to his points noted previously about the importance of moving towards open models of innovation and a changed role for funders, by considering the game changing impact of emerging technologies (such as personalized healthcare) on the Innovation Pipeline (Session 7a). He argued: *“These technologies wrap around innovation in unique and cumulative ways, which accelerates the initiatives in the pipeline towards impact. The role of the funder and the funding models will need to permit the collisions to occur, to embrace new, open models of innovation”* (7Aiii).

Fraser described the various steps along the Innovation Pipeline from the perspective of the health care system. The initial steps of collectively identifying care gaps in Alberta Health Services, and identifying or funding research to address them, were described in Component Two, but remaining steps are outlined here. In Step 3, she stated: *“we ‘test’ what we have developed; funding sources serve as an enabling function to help move work down the pipeline shown in* [Fig. 2]*. What is learned at each stage of the pipeline circles around and informs the ‘science’”* (7Bii). In Step 4 proven innovations are spread to scale, and in Step 5 they are implemented and sustained in care. Her final Key Message reflected this, and highlighted a contrast with the earlier steps when researchers had more involvement: *“As work moves to adoption and sustainability within the health system the clinical and operational leaders assume more of a lead to ensure the sustainability of the gains that have been discovered and are now being used”* (7Biv).

The specific example Gregg Nelson provided in his presentation (Session 7C) strengthened the general accounts from the Albertan team. He showed how the success of the Enhancing Recovery After Surgery (ERAS) approach in Alberta could be described in terms of the five steps in the AHS Innovation Pipeline (Fig. 2). The Surgery SCN identifies care gaps as the first step. For the second step of identifying evidence, or funding research to produce it, the SCN draws on the work the multi-professional international ERAS Society. The work of the Society is aimed at developing perioperative care and improving recovery, and in one analysis, had been shown to reduce length of stay by 2.5 days and reduce complications by 50%. For Step 3, the *“Implementation ‘Test’ in Alberta”*, the Surgery SCN received a PRIHS grant from AI (formerly Alberta Innovates – Health Solutions) in 2013 to apply the approach to colorectal surgery in Alberta. This proved successful [32] and they proceeded to Step 4, *“Implementation (Work to Scale)”* in which they received further funding from 2016 to 18 to spread ERAS to additional locations and care pathways. The Surgery SCN have now reached Step 5, *“Implemented and Sustained in Care”*, with Nelson stating: *“This started as a proof of concept project in colorectal surgery and since that time has evolved into a province-wide operationalized program now in multiple surgical areas and multiple hospitals”* (7Ciii). This case study provides a good example of how a global evidence-based approach was implemented locally with additional funding to develop and apply the approach in the AHS.

Finally, in session 7, Mrklas and Flynn’s analysis of the role of the pipeline highlights: *“The importance of both the pipeline to orient change, investments in change, intent to scale-spread, sustain, and make impact, and of co-design in healthcare settings”* (7Ei). Additionally, they went on to stress that: *“Implementation and sustainability have much in common – recognize the importance of sustainability in the pipeline to impact, and the need for research to fill sustainability knowledge gaps”* (7Eii).

In this component, we have seen both accounts of how long it takes to build up a system to encourage *Implementation to Impact* and attempts to speed up the processes from research to implementation and impact. One of Ovseiko’s insights as moderator of session 9 possibly provides a way to bring these concerns together to some degree. He referred to the “‘*Push the Pace’ approach at NIHR to continuously improve its processes”* (9E). This project was an attempt to speed up the research and implementation processes through the NIHR examining its own processes and making improvements, initially as a one-off but with the intention that it should become a process of continuous improvements (see Moran et al., 2019 [33] for a fuller account). Other presenters also referred to continuous improvement approaches, including Elizabeth Shirt who included continuous improvement in the third element of her approach, i.e. as part of the Mindset (2Ciii). As with the Push the Pace initiative, continuous improvement approaches are likely to operate at a systems level.

## Section two: discussion of emerging themes and challenges

### Developing comprehensive and coherent systems promoting implementation to impact

Contributions made at the Summit support the view that globally there is increased interest in the benefits that can accrue from adopting a systems approach to (health) R&I. This was seen in particular, but not only, in the presentations at the International Summit from five large funders at national or regional levels, spread over three continents: AI, AQuAS, CSIRO, NIHR and ZonMw. In each case, the presentations from delegates from these organizations described activity, and progress, associated with *Implementation to Impact* across various components listed in the WHO framework [9]. This illustrates that when the vision, stakeholder engagement in agenda-setting (supported by appropriate ethical considerations), M&E, availability of resources and capacity building are all aligned and oriented towards producing and implementing robust and relevant evidence, then sustained innovation and impact seem more likely to be achieved. These funders are all moving towards some of the key desirable characteristics of a successful health research system that were identified in a recent review for WHO [10].

Several presentations stressed that the existence of an impact culture was vital in achieving sustainable impact, but it is probable that such a culture is likely to be fostered when the various components are all aligned towards achieving sustainable impact. For example, if the M&E, or assessment, of the research includes a focus on rewarding efforts to implement research and generate impacts, then this provides both incentives and a justification for spending time on such activities, which in turn will help foster and sustain an impact culture. The elements become mutually reinforcing and illustrate how appropriate structures can work positively with the human factors. The presentations in session 3 (John, Radó-Trilla, Keenan) and 9D (Hough and Kamenetzky) illustrate how whole systems could be geared up towards an impact culture. Keenan elaborated on how some of the ways of embedding an impact culture in the research organization could be top-down, including the investment decisions and the evaluation approaches. Others could be bottom-up, for example, co-developing impact pathways and monitoring plans with key stakeholders, as well as developing positive relations with users (3C). The capacity building component of a research system could also be seen as a bottom up approach to developing an impact culture.

Such presentations, plus others from organizations such as AI and NIHR, also illustrated the enthusiasm, even inspiration, which can be generated by working collaboratively within an organization adopting a systems approach to promote *Implementation to Impact.* This was seen perhaps most clearly in the series of presentations in session 7, *Accelerating Scale and Spread for Sustainable Impact – A Local Approach.* Each presentation focused on different aspects of the Innovation Pipeline shown in Fig. 2. The cumulative effect of the series of diverse presentations on a shared approach was especially powerful, as also reflected in the first action recommended by Crelinsten: *“promote the use of the pipeline concept and provide training on its use to key stakeholders”* (7F).

### Embedding the research system into the (healthcare) user system: feasibility and supporting implementation to impacts

Those R&I funders that have gone furthest in developing a research system to promote *Implementation to Impacts,* also often seem to be pioneering attempts to build the research system into user communities or systems. Keenan (Session 3C), for example, described how in Australia CSIRO are attempting to do this across the range of fields in which they operate. It is particularly noticeable, however, as a feature of the pioneering work of the health R&I systems and the relevant healthcare systems highlighted at the Summit.

This combined approach in relation to the health field was most clearly described in the accounts above of several presentations in Summit session 7 on the Innovation Pipeline, including Fraser’s account of all members of the PRIHS project being part of a network *“coming together to solve problems and advance care”* (7Bi). Zimmermann’s account of the KT Platform established in Alberta by the AbSPORU explicitly stated the “*Implementation Science (IS) labs are research teams embedded into the health care system to conduct studies using real world data to inform best practices”* (7Dii). Furthermore, as noted in the discussion in session 7, Cy Frank, when directing AI’s health research, served on the Advisory Committee of the NIHR-funded review of the global literature on the improved healthcare performance of research active healthcare organizations. It was reported in 2013, but the related article was Boaz, et al., 2015 [34]. Such evidence on the benefits of research active healthcare organizations was compatible with Frank’s own initiatives in Alberta.

McMahon’s account of the CIHR-IHSPR’s Health System Impact Fellowship program (Session 5A) illustrates the increasing efforts across Canada to build research into healthcare systems. The program was, in part, a response to the emergence of *“learning health systems”* (5Ai). Early findings from the evaluation of the program have found *“Health system organizations are keen to embed PhD talent as part of their teams, and PhD trainees and post-docs are keen for impact-oriented training opportunities”* (5Aiii).

### Addressing the challenges to implementation to impact and tensions identified

In addition to discussion of promising practices in implementation and impact, Summit deliberations also explored key challenges and tensions facing *Implementation to Impact*, and the role of a systems approach in facilitating some ways to address them. Key examples of the challenges and tensions are analysed below.

- In various presentations, issues were raised about how to reduce the many years often taken to achieve implementation and realize impact in R&I. As was seen in the presentations from session 7, the drive to accelerate *Implementation to Impact* was often associated with attempts to take coordinated action aimed at various components of the system. For example, engaging stakeholders throughout the processes is more likely to generate the circumstances in which the R&I system meets the needs of the local user system. Concerns were raised, however, in other sessions about how far the resources would readily be available to fund the activities within the system designed to build the internal capacity and structures necessary to promote innovation and impact. Furthermore, while presentations such as those from Kamenetzky (1C) and Keenan (3C) showed a keenness to promote greater engagement and impact, they also recognized that developing the structures, processes and relationships to do this could take research funding organizations considerable time.
  Additionally, Reijmerink from ZonMw, which has made considerable efforts to improve implementation and impacts, made the usefully realistic point in session 9C, as noted above, about the *“accumulation of small wins”* (9Ciii). This illustrates the point that there might be a need to negotiate between “speed” and “timeliness”. Similarly, lessons come from a detailed exploration of how to analyse the time taken from initial research to translation into improved policies and care [7]. The various case studies analysed reveal enormous complexities, and also differences in the opportunities to reduce the time. This reinforces the need for careful analysis and adaptive strategies when developing plans to speed up the processes of research and implementation. Additional workloads often lead to increased tensions.
- Challenges and tensions arise from the inevitable resistance to change. In this report we are primarily describing what was said about how the R&I system itself might be able to address such challenges. Various aspects of the challenges in organizations in which innovations are being implemented might be beyond the R&I system to address. (Nevertheless, the more firmly the research system is embedded into the user organization, perhaps as with AI and AHS in Alberta, the better). More pertinent to our analysis is the resistance that might arise in the R&I system itself to changes related to developing some of the components necessary in a system geared up to emphasize *Implementation to Impact*. Various presenters, including Hanney (1B) and Radó-Trilla (3B), gave examples of such resistance. Here, the various points made above about careful attention to developing an impact culture could be helpful in reducing the tensions.
- Coproduction in R&I is one area where resistance to change is, at least in part, based on genuine difficulties in developing new approaches. Ovseiko’s opening Key Note presentation drew attention to the important issues raised in a recent article by Oliver et al.: *The dark side of coproduction: do the costs outweigh the benefits for health research?* Oliver et al. (2019) offer advice as to when and how to consider coproduction [35]. Such issues are probably best analysed within a systems approach.
- As noted by Reijmerink, it is important to focus on the quality of the research production before considering implementing the resulting findings: *“Don’t talk about impact when the underlying evidence is not robust in terms of relevance and quality”* (9 Ci). So, in addition to the general challenge to research funders and organizations to avoid the previously reported high rate of research waste, there are particularly acute concerns when the evidence produced is being considered for implementation. Not necessarily every aspect associated with research waste could be addressed by developing strong research systems. Nevertheless, many of the actions described above as fitting into a system would improve the quality of research. These include the efforts to identify research agendas relevant to the needs of users, appropriate research ethics procedures, and developing the capacity of researchers. Again, however, some of the steps to improve quality will require additional resources and time.
  As also noted, Reijmerink’s presentation introduced the Consensus Statement from the EViR Funders’ Forum which sets out approaches to improve research quality. Interestingly, aspects of the work of all three of the health research funding bodies that created and co-convene the EViR Forum were described at the Summit. While only the presentation from Reijmerink based at ZonMW specifically mentioned the Forum (Session 9C), the other two funders were NIHR, several of whose other initiatives were described, and PCORI, whose work on stakeholder engagement was described in Gerlach’s presentation (Session 4C). This might illustrate that well-organized research funders adopting a systems approach tend to be interested in addressing a range of challenges. They might also adopt a leading role as credible change-makers in value-driven knowledge ecosystems.
- The importance of ensuring the robustness of the research before attempting to achieve impact by implementing the findings, links to other challenges and tensions around the balance between use of local or global research. The website of the EViR Funders’ Form sets out some principles behind the four points in the Consensus Statement described above. The second principle states: *“Research should only be funded if set in the context of one or more existing systematic reviews of what is already known or an otherwise robust demonstration of a research gap”* [29]. This, therefore, sets a high premium on drawing on the global stock of knowledge, where it is available. The approach described in session 7 with the Innovation Pipeline seems broadly in keeping with this principle. Generally, however, it is not always clear that all researchers and implementation teams give sufficient attention to the global stock of knowledge before starting studies, or implementing the findings, respectively. The aim should be to develop systems that invest in the capacity to review and draw on the international literature, as well as in mechanisms to promote the *Implementation to Impact* of their own research where appropriate.

## Section three: lessons and further work

The overall lesson from the Summit is that various organizations in Canada and internationally have made considerable progress on *Implementation to Impact,* often as a result of well-planned initiatives*.* This has particularly been the case when the organizations have been, in the words of Murphy (7A), *“value-hunters”* rather than “*fund and forgetters”*, adopted a systems approach and undertaken action in a coherent way across a range of components of a research system. There are, of course, many further examples from the wider international community, than those presented at the Summit. Some of those were presented at the earlier *In the Trenches: Research Translation for Health Impact International Symposium* at Oxford in 2018 [3, 4], including work from the Novo Nordisk Foundation in Denmark, which has produced a series of reports on the wider impact of the research it funds [36, 37]. Nevertheless, in every system there are still many challenges and tensions.

Discussion of further work falls into two categories.

- First, recommendations are made based on lessons from the Summit as to how R&I funders should best organize themselves to continue to undertake activities promoting *Implementation to Impact.*
- Second, thoughts about how the *In the Trenches* team might continue to encourage and support developments in this field.

### How might research and innovation funders best organize themselves to continue to undertake activities promoting implementation to impact

Diverse recommendations can be made in relation to aspects of individual components of a (health) research system in order to promote *Implementation to Impact.* These components were all covered at the Summit, and include: engaging stakeholders in agenda-setting and later stages of R&I; developing appropriate impact assessment approaches; equitably building human capacity to conduct, absorb and use research and organize research systems, and appropriately drawing on infrastructure funding and databases; producing valid research outputs; and implementing evidence in ways that lead to sustainable impact. The Actionable Insights recorded in Additional file 3 from each of the sessions cover all these points to varying degrees, along with additional ideas such as the importance of an impact culture in enhancing the chances of achieving sustainable innovation and impact.

It seems sensible to make an overall recommendation that the more that funders and organizations can work on these issues in a comprehensive and coherent way, the better the chances of addressing the considerable challenges and tensions that remain. Furthermore, embedding such research systems into the user system (e.g. healthcare system) is likely to provide the most productive opportunities for stakeholder engagement and *Implementation to Impact.* The most comprehensive approach described in detail at the Summit was that in Alberta with the Innovation Pipeline. As noted, Crelinsten, the moderator of session 7 in which Alberta’s Innovation Pipeline was presented, observed in his Actionable Insights that: *“The pipeline is both a reflective tool and a line of sight that can be used to regularly embed the creation and use of high quality evidence for impact”* (7F). He then made his recommendation to: *“promote the use of the pipeline concept”* (7F).

As we also saw from the Summit presentations, some funders are already making considerable progress in applying a systems approach. While the overall progress made by the NIHR was not specifically considered at the Summit, analysis of its approach could provide crucial further evidence for any recommendation to adopt a comprehensive and coherent approach. Sally Davies analysed the achievements of the first 10 years of the NIHR that she created, and while this was not specifically about *Implementation to Impact*, it did illustrate the NIHR had made progress on many of the components that constitute an effective research system [38]. There was also a rather more specific, but briefer, analysis considering the considerable progress made by the NIHR on most of the components of the WHO’s research system framework [10, 39]. These analyses were informed by a major assessment of the achievements of the NIHR during its first 10 years [40].

If the thinking in these proceedings about the components that constitute a system was combined with something along the lines of the AHS Innovation Pipeline, it might possibly form the basis for a new framework. This is an idea that could be taken further forward.

### Future in the trenches resources and events

In the post-Summit evaluation survey several respondents expressed an interest in more practical information on implementing the ideas presented at the Summit. One response is that some practical guides relevant to points made in the Summit have been outlined here in the proceedings and added to the Resources section of the Summit website, even in some cases when they were not specifically referenced in the presentations. Examples of such material, presented in the order of the research systems framework, include:

- Ways to engage stakeholders in research, including in agenda-setting [14, 15, 41]
- Ethical considerations for patient and public involvement [17]
- Ways to assess research impact and apply research impact frameworks [1, 20–23]
- Building capacity [24, 25, 42–47]
- Producing valid research outputs [29]
- Implementation to Impact (Fig. 2) [16]
- Continuously improving the (health) research system [30, 33].

The International School for Research Impact Assessment (ISRIA) continues to demonstrate its value as it becomes an increasingly important source for analysts exploring practical ways in which organizations implement impact assessment [22]. Therefore, a further response to the interest expressed in the Summit evaluation for more information has been to consider the possibility of building on the experience of the ISRIA to run international short courses on *Implementation to Impact*. Any updates on this idea will be circulated to interested parties and disseminated via the Summit website.

## Conclusions

Contributions made at the Summit support the view that globally there is increased interest in the benefits that can accrue from adopting a systems approach to (health) R&I. The overall lesson from the Summit is that various organizations in Canada and internationally have made considerable progress on *Implementation to Impact*, often a result of well-planned initiatives. Two of the key learnings to emerge at the Summit partly highlight the value of the efforts devoted to creating an engagement platform for diverse stakeholders to share emerging knowledge and practical experiences: 1) collaboration between researchers and potential users, and 2) the adoption by funders of approaches involving an increasing range of responsibilities and activities. These were all intended to increase successful implementation and achievement of impact. The post-Summit evaluation survey results reinforced delegates’ interest for more practical information on implementing some of the ideas presented at the Summit. The Summit website (https://inthetrenchessummit.com/) will be periodically updated with new resources and information about future *In the Trenches* events.

## Supplementary information

**Additional file 1.** Summit’s full program.

**Additional file 2.** List of members of the Summit’s International Organizing Committee and the Alberta Innovates Advisory Committee.

**Additional file 3. Table 1.** Key Messages (from presentations) and Actionable Insights (by session)

## Data Availability

The materials and information used in the preparation of these proceedings are readily available at the Summit website (https://inthetrenchessummit.com/).

## Abbreviations

AbSPORU: Alberta Strategy for Patient Oriented Research SUPPORT Unit
AHS: Alberta Health Services
AI: Alberta Innovates
AQuAS: Agency for Health Quality and Assessment of Catalonia
CAHS: Canadian Academy of Health Sciences
CFI: Canada Foundation for Innovation
CIHR: Canadian Institutes of Health Research
CIHR-IHSPR: Canadian Institutes of Health Research – Institute of Health Services and Policy Research
CSIRO: Commonwealth Scientific and Industrial Research Organization
ERA: Emissions Reduction Alberta
ERAS: Enhancing Recovery After Surgery
EViR: Ensuring Value in Research
ISRIA: International School on Research Impact Assessment
KT: Knowledge Translation
M&E: Monitoring and evaluating
NIHR: National Institute for Health Research
OBI: Ontario Brain Institute
PaCER: Patient and Community Engagement Research
PCORI: Patient-Centered Outcomes Research Institute
R&I: Research and Innovation
SARIS: Health Research and Innovation Assessment System
SCN: Strategic Clinical Network
TRL: Technology Readiness Levels
UK: United Kingdom
USA: United States of America
WHO: World Health Organization
ZonMw: Netherlands Organization for Health Research and Development
