# Supplementary material for: A systems approach for optimizing implementation to impact: meeting report and proceedings of the 2019 In the Trenches: Implementation to Impact International Summit
Source: BMC Proc. 2020 Jul 24;14(Suppl 6):10. doi: 10.1186/s12919-020-00189-x (PMC7379765; doi:10.1186/s12919-020-00189-x)
Supplement: Supplementary file 1 — Additional file 1. Summit’s full program. [file 12919_2020_189_MOESM1_ESM.pdf]

In the Trenches:
Implementation to Impact International Summit

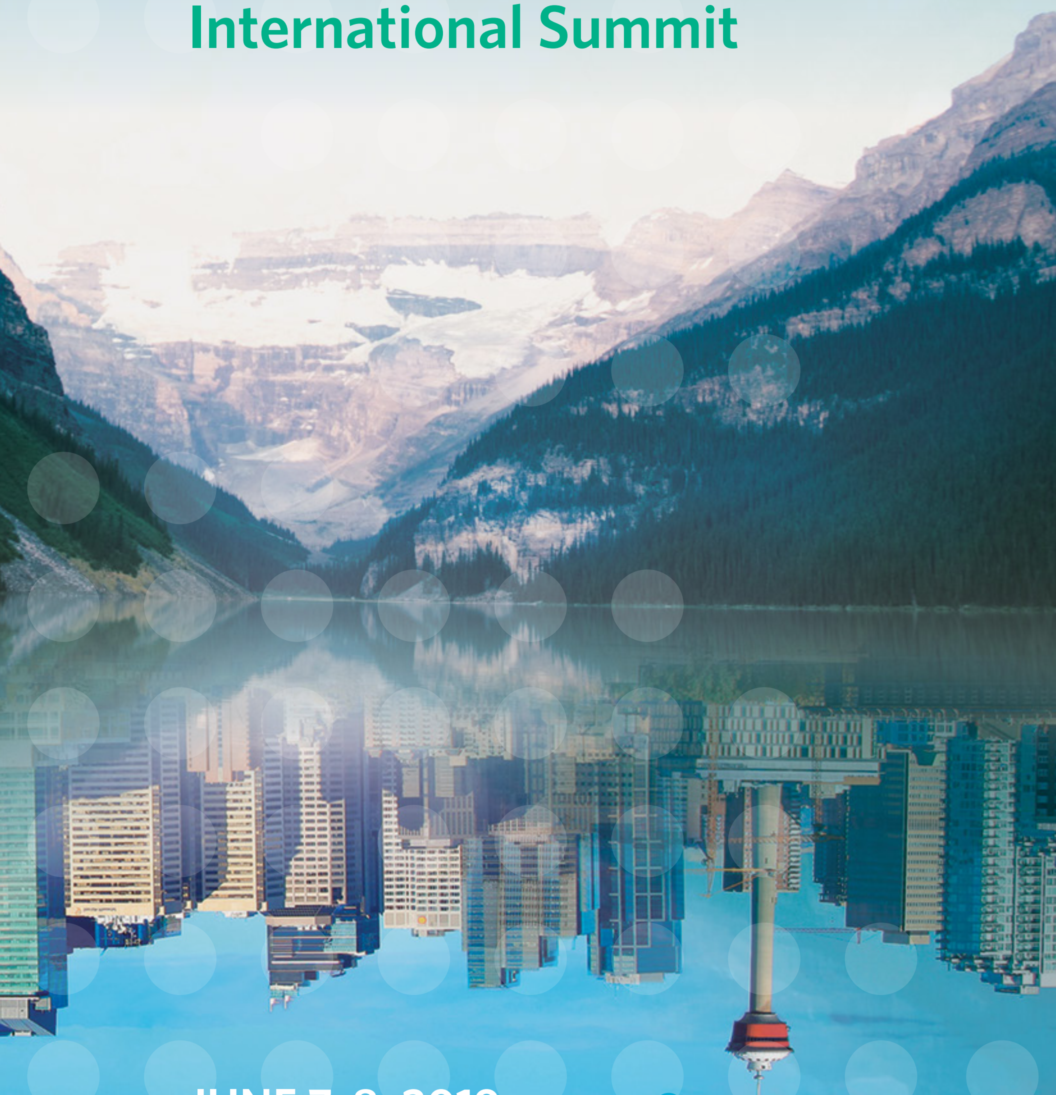

JUNE 7–8, 2019
BANFF, ALBERTA, CANADA

@ABInnovates #implementation2impact
Alberta Innovates
albertainnovates.ca

WELCOME MESSAGE

Welcome to the In the Trenches: Implementation to Impact International Summit 2019!

Research and innovation (R&I) are the engine of sustainable social well-being and economic growth. We are so pleased to bring together this group of experts, researchers, policy makers, and entrepreneurs to discuss the leading-edge perspectives and practices that enable us to maximize the impacts of our innovations.

This is the second iteration of the "In the Trenches" series, a partnership of international funders and organizations that is co-hosted by Alberta Innovates and the University of Oxford. We have come together to share the latest research and approaches for implementing R&I to maximize societal impact locally, nationally, and globally.

Thank you for joining us and we look forward to your contributions over the next day and a half! We hope you will be inspired with new strategies and ideas that will invigorate your own scale and spread initiatives.

Kathryn Graham
Alberta Innovates

Pavel Ovseiko
Oxford University

SUMMIT CO-DIRECTORS

PROGRAM AGENDA

DAY 1

FRIDAY, JUNE 7, 2019

2:00 PM – 3:00 PM

Kinnear Centre for Creativity & Innovation (KC): KC100 Galleria

Registration Check-in

3:00 PM – 3:30 PM

KC 105

Welcome Address

Tim Murphy (Alberta Innovates, CAN)

Introduction to the "In the Trenches" Impact Summit Series

Kathryn Graham (Alberta Innovates, CAN) &
Pavel Ovseiko (University of Oxford, UK)

Getting to Know Your Neighbour in the Trenches

Maxi Miciak (Alberta Innovates, CAN)

Theme: Setting the Stage for Implementation to Impact (and Back Again)

Implementing research and innovation (R&I) to achieve impact is contested and complex, with unpredictable use of results. The purpose of this theme is to introduce participants to frameworks and approaches that make sense of the complexity to facilitate implementation to impact.

3:30 PM – 4:45 PM

KC 105

Session 1: Frameworks and Approaches to Navigate Implementation to Impact

Panellists will introduce different principles, frameworks and approaches that acknowledge and make sense of complexity through the pathways from implementation to impact. The session will end with applications in practice.

- Complexity, implementation science and value co-creation
- Impact Frameworks in action
- The view from within: learning from other research funders by asking 'how do you do it?'

MODERATOR: Pavel Ovseiko (University of Oxford, UK)

PANELLISTS: Pavel Ovseiko (University of Oxford, UK)
Stephen Hanney (Brunel University London, UK)
Kathryn Graham (Alberta Innovates, CAN)
Adam Kamenetzky (NIHR, UK)

4:45 PM – 5:30PM

Session 2: Flipping the Switch – Cross-sector Learnings from Near and Far

KC 105

Local, national and international Panellists from sectors outside of health will convey their experiences and perspectives on implementing scale and spread initiatives to achieve societal benefit.

- Clean technology the Alberta way
- Scale-and-spreading infrastructure nationally
- Business innovation the Australian way

MODERATOR: Dale Sanders (Health Catalyst, US)

PANELLISTS: Elizabeth Shirt (ERA, CAN)
Laura Hillier (CFI, CAN)
Peter Riddles (CSIRO, AUS)

5:30 PM – 6:00 PM

Transportation to Banff Springs Hotel Networking Reception

6:00 PM – 7:30 PM

Cocktails and Refreshments: Networking Reception

Fairmont Banff Springs Hotel: Upper Valley Bow Terrace

DAY2

SATURDAY, JUNE 8, 2019

7:00 AM – 8:00 AM

Breakfast

Banff Centre Vistas Dining Room (Sally Borden Building)

8:00 AM – 8:15 AM

Overview of the Day

KC 105

Kathryn Graham (Alberta Innovates, CAN) &
Pavel Ovseiko (University of Oxford, UK)

Theme: Creating a Culture for Societal Impact

Effective implementation requires organizations and ecosystems to be aware of the structural and social influences necessary for successful innovative and long-term integration of R&I to achieve societal impact. Panellists will present and discuss ways they have contributed to creating a culture for societal impact.

8:15 AM – 9:00 AM

Session 1: Building Stakeholder Engagement Platforms for Culture Change

KC 105

Panellists will review stakeholder engagement approaches they have designed and implemented to affect culture change for optimizing use of R&I.

- Creating an organizational culture to move implementation to impact
- Engaging stakeholders in the development of a system of Health Research Assessment (SARIS) in Catalonia
- The importance of planning for effective adoption pathways to deliver social and economic impact

MODERATOR: Peter Riddles (CSIRO, AUS)

PANELLISTS: Reesa John (Alberta Innovates, CAN)
Núria Radó-Trilla (AQuAs, Spain)
Tom Keenan (CSIRO, AUS)

9:00 AM – 9:45 AM

Session 2: Switching to Practice – Integrating Patients' Perspectives on What Matters

KC 105

Panellists will introduce real-world approaches for optimizing patients' contributions to inform what needs to be assessed in implementation and impact initiatives.

- Meaningful participation of patients in R&I for implementation and impact
- Importance of patient engagement with scale and spread of research and innovations into the health system
- Engaging patients throughout the research lifecycle

MODERATOR: Tim Murphy (Alberta Innovates, CAN)

PANELLISTS: Jean Miller (Patient Representative, (PaCER), Alberta, CAN)
Jordan Antflick (Ontario Brain Institute, CAN)
Lauren Gerlach (AcademyHealth, US)

9:45 AM – 10:15 AM

Refreshment and Networking Break and Dialogue with Presenters

10:15 AM – 11:15 AM

Session 3: Building a Diverse Talent Pool for the Future

KC 105

Panellists will speak about innovative ways of diversifying and strengthening the talent pool of tomorrow to bridge the gap between research and innovation and impact.

- Canadian health system impact fellowships
- Talent for the workforce of tomorrow
- Gender equity pathway to impact
- Women's leadership and advancement in 20 Catalan biomedical research centers

MODERATOR: Adam Kamenetzky (NIHR, UK)

PANELLISTS: Meghan McMahon (CIHR-IHSPR, CAN)
Alex Clark (University of Alberta, CAN)
Pavel Ovseiko (University of Oxford, UK)
Eduard Güell (AQuAS, Spain)

11:15 AM – 12:00 PM

WORLD CAFÉ (concurrent sessions)

Session 1: Connecting the Dots Between the Innovation Process and Societal Benefit

KC 105

Participants will engage in dialogue centred on unpacking the connection between the innovation process with economic and social development or a "systems" approach to innovation achieving impact.

MODERATOR: Peter Riddles (CSIRO, AUS)

Session 2: Addressing Sustainability in Real-world Applications

KC 206

Participants will engage in focused conversation on navigating the quagmire of sustaining implementation efforts in real-world applications.

MODERATORS: Kelly Mrklas (AHS, CAN)
Rachel Flynn (University of Alberta, CAN)

12:00 PM – 1:00 PM

Lunch

Vistas Dining Room

Theme: Novel Approaches for Scaling Research and Innovation to Impact

Implementing R&I in isolated settings is one thing – appropriately scaling innovation beyond 'the pilot' is another. Panellists will illustrate key considerations and creative ways they have approached scaling innovations in their contexts.

1:00 PM – 2:15 PM

Session 1: Accelerating Scale and Spread for Sustainable Impact – A Local Approach

KC 105

Panellists will review innovative ways they have scaled-up and spread initiatives in the real-world, conveying practical approaches for sustaining impact.

- Strategic considerations for research and innovation scale and spread
- Co-creation approach for value - Partnership for Research and Innovation in the Health System (PRIHS)
- Enhanced Recovery After Surgery (ERAS) - the Alberta Experience
- Using implementation labs to clear a path to impact
- Sustaining implementation efforts leveraging impact assessment

MODERATOR: Jeffery Crelinsten (Re$earch Money, CAN)

PANELLISTS: Tim Murphy (Alberta Innovates, CAN)
Nancy Fraser (Critical Care Strategic Clinical Network, AHS, CAN)
Gregg Nelson (University of Calgary, CAN)
Gabrielle Zimmermann (AbSPORU KT Platform, CAN)
Kelly Mrklas (AHS, CAN) & Rachel Flynn (University of Alberta, CAN)

2:15 PM – 2:45 PM

Refreshment and Networking Break and Dialogue with Presenters

2:45 PM – 3:30 PM

Session 2: Show Me the Data

KC 105

Panellists will discuss data strategies and analytics that address data use in complex and dynamic environments.

- Data-driven insights for accelerating scale-up and spread
- Semantic technologies and ontology-based data access for research impact monitoring and evaluation
- Navigating the Canadian data landscape

MODERATOR: Dale Sanders (Health Catalyst, US)

PANELLISTS: Dale Sanders (Health Catalyst, US)
Alba Velasco (SIRIS Academics, Spain)
Rick Glazier (CIHR-IHSPR, CAN)

3:30 PM – 4:30 PM

Session 3: Engaged Assessment for Sustaining Impact

KC 105

Panellists will discuss ways of engaging stakeholders in measuring, assessing and improving impact at local, national, and international levels.

- Social economic return on investment
- Using a co-creation and layered approach to scaling and spreading impact assessment Canada
- Maximizing sustainable impact in The Netherlands
- Designing a monitoring, evaluation and learning approach to maximise sustainable impact for the UK National Institute for Health Research (NIHR)'s Global Health portfolio

MODERATOR: Pavel Ovseiko (University of Oxford, UK)

PANELLISTS: Alan O'Connor (RTI, US) & Kathryn Graham (Alberta Innovates, CAN)
Maxi Miciak (Alberta Innovates, CAN)
Wendy Reijmerink (ZonMw, Netherlands)
Adam Kamenetzky & Carrie Hough (NIHR, UK)

SATURDAY, JUNE 8, 2019

DAY2

4:30 PM – 5:00 PM

KC 105

Reflections and Summary
Stephen Hanney (Brunel University London, UK)

Next Steps in the Series
Kathryn Graham (Alberta Innovates CAN) &
Pavel Ovseiko (University of Oxford, UK)

Farewell Address
Laura Kilcrease (Alberta Innovates, CAN)

5:00 PM – 6:00 PM

Maclab Bistro

Closing Connections and Dialogue with Presenters & Participants

ACKNOWLEDGMENTS

PROGRAM CO-DIRECTORS

Kathryn Graham, Alberta Innovates, CAN

Pavel Ovseiko, University of Oxford, UK

INTERNATIONAL ORGANIZING COMMITTEE

Paula Adam-Bernard, AQuAS, Spain

Mark Taylor, NIHR, UK

Gert Balling, Novo Nordisk Foundation, Denmark

Tom Keenan, CSIRO, Australia

Wendy Reijmerink, ZonMW, Netherlands

Stephen Hanney, Brunel University London, UK

Megan Collado, AcademyHealth, US

Maxi Miciak, Alberta Innovates, CAN

ALBERTA INNOVATES ADVISORY COMMITTEE

Laura Kilcrease

Rollie Dykstra

Tim Murphy

Lyn Brown

PANELLISTS & MODERATORS

Adam Kamenetzky (NIHR, UK)

Alan O'Connor (RTI, US)

Alba Velasco (SIRIS Academics, Spain)

Alex Clark (University of Alberta, Canada)

Carrie Hough (DHSC, UK)

Dale Sanders (Health Catalyst, US)

Eduard Güell (AQuAS, Spain)

Elizabeth Shirt (ERA, Canada)

Gabrielle Zimmermann (AbSPORU KT Platform, Canada)

Gregg Nelson (University of Calgary, Canada)

Jean Miller (Patient and Community Engagement Research (PaCER), Alberta, Canada)

Jeffery Crelinsten (Re$earch Money, Canada)

Jordan Antflick (Ontario Brain Institute, Canada)

Kathryn Graham (Alberta Innovates, Canada)

Kelly Mrklas (AHS, Canada)

Laura Hillier (CFI, Canada)

Lauren Gerlach (AcademyHealth, US)

Maxi Miciak (Alberta Innovates, Canada)

Meghan McMahon (CIHR-IHSPR, Canada)

Nancy Fraser (Critical Care Strategic Clinical Network, AHS, Canada)

Núria Radó-Trilla (AQuAS, Spain)

Pavel Ovseiko (University of Oxford, UK)

Peter Riddles (CSIRO, Australia)

Rachel Flynn (University of Alberta, Canada)

Reesa John (Alberta Innovates, Canada)

Rick Glazier (CIHR-IHSPR, Canada)

Stephen Hanney (Brunel University London, UK)

Tim Murphy (Alberta Innovates, Canada)

Tom Keenan (CSIRO, Australia)

Wendy Reijmerink (ZonMw, Netherlands)

LIST OF ORGANIZATIONS AND ABBREVIATIONS

AcademyHealth, United States (US)

Agency for Health Quality and Assessment of Catalonia (AQuAS), Spain

Alberta Health Services (AHS), Canada (CAN)

Alberta Strategy for Patient Oriented Research Support Unit (AbSPORU) Knowledge Translation (KT) Platform, Canada (CAN)

Alberta Innovates, Canada (CAN)

Brunel University London, United Kingdom (UK)

Canadian Foundation for Innovation (CFI), Canada (CAN)

Canadian Institute of Health Research – Institute of Health Services and Policy Research (CIHR-IHSPR), Canada (CAN)

Commonwealth Scientific and Industrial Research Organisation (CSIRO), Australia

Department of Health and Social Care (DHSC), United Kingdom (UK)

Emissions Reduction Alberta (ERA), Canada (CAN)

Health Catalyst, United States (US)

National Institute for Health Research (NIHR), United Kingdom (UK)

Ontario Brain Institute, Canada (CAN)

Patient and Community Engagement Research (PaCER), Canada (CAN)

Re$earch Money, Canada (CAN)

RTI International, United States (US)

SIRIS Academics, Spain

University of Alberta, Canada (CAN)

University of Calgary, Canada (CAN)

University of Oxford, United Kingdom (UK)

ZonMW, Netherlands

PARTNERS

COLLABORATORS

In the Trenches:
Implementation to Impact International Summit

JUNE 7–8, 2019
BANFF, ALBERTA, CANADA
