# Supplementary material for: A systems approach for optimizing implementation to impact: meeting report and proceedings of the 2019 In the Trenches: Implementation to Impact International Summit
Source: BMC Proc. 2020 Jul 24;14(Suppl 6):10. doi: 10.1186/s12919-020-00189-x (PMC7379765; doi:10.1186/s12919-020-00189-x)
Supplement: Supplementary file 2 — Additional file 2. List of members of the Summit’s International Organizing Committee and the Alberta Innovates Advisory Committee. [file 12919_2020_189_MOESM2_ESM.pdf]

Members of the International Organizing Committee

- Kathryn E. A. Graham, Summit Co-Director, Executive Director, Performance Management and Evaluation, Alberta Innovates, Canada
- Pavel V. Ovseiko, Summit Co-Director, Senior Research Fellow in Health Policy and Management, Radcliffe Department of Medicine, University of Oxford, UK
- Heidi Chorzempa, Director (Interim), Performance Management and Evaluation, Alberta Innovates, Canada
- Maxi Miciak, Cy Frank Fellow in Impact Assessment, Alberta Innovates, Canada
- Stephen R. Hanney, Emeritus Professor, Health Economics Research Group, Brunel University London, UK
- Megan Collado, Director, AcademyHealth, US
- Wendy Reijmerink, Senior Staff Member, Strategy and Innovation, Netherlands Organization for Health Research and Development (ZonMw), Netherlands
- Tom Keenan, Senior Advisor, Planning Performance and Evaluation, Commonwealth Scientific and Industrial Research Organization (CSIRO), Australia
- Mark Taylor, Head of Impact, National Institute for Health Research (NIHR), UK
- Gert V. Balling, Senior Scientific Officer, Novo Nordisk Foundation, Denmark
- Paula Adam, Director of Research, Agency for Health Quality and Assessment of Catalonia (AQuAS), Spain

Members of the Alberta Innovates Advisory Committee

- Laura Kilcrease, CEO, Alberta Innovates, Canada
- Rollie Dykstra, Vice President, Investments, Alberta Innovates, Canada
- Tim Murphy, Vice President, Health, Alberta Innovates, Canada
- Lyn Brown, Vice President, Marketing and Communications, Alberta Innovates, Canada
