# Supplementary material for: A systems approach for optimizing implementation to impact: meeting report and proceedings of the 2019 In the Trenches: Implementation to Impact International Summit
Source: BMC Proc. 2020 Jul 24;14(Suppl 6):10. doi: 10.1186/s12919-020-00189-x (PMC7379765; doi:10.1186/s12919-020-00189-x)
Supplement: Supplementary file 3 — Additional file 3. Table 1. Key Messages (from presentations) and Actionable Insights (by session) [file 12919_2020_189_MOESM3_ESM.pdf]

A systems approach for optimizing implementation to impact: meeting report and proceedings of the 2019 *In the Trenches: Implementation to Impact International Summit*

Additional File 3. Table 1: Key Messages (from presentations) and Actionable Insights (by session)

Table 1 lists the Key Messages supplied by each presenter in the order in which the presentations were given, and the Actionable Insights from the moderator of each session. Light editing was undertaken to present these in a reasonably consistent fashion, and the numbering used for each Key Message and Actionable Insight is then used for referencing extracts from them in the analysis provided throughout sections 1-3 of the Proceedings report.

Table 1.

THEME AND SESSION
**THEME ONE: SETTING THE STAGE FOR IMPLEMENTATION TO IMPACT (AND BACK AGAIN)**
Session 1: Frameworks and Approaches to Navigate Implementation to Impact<br><br>**Panelists introduced different principles, frameworks and approaches that acknowledge and make sense of complexity through the pathways from implementation to impact. The session ended with applications in practice.**<br><br>**A) Complexity, implementation science and value co-creation: Pavel Ovseiko (University of Oxford, UK)**<br>*i) Implementation to impact is more of an art than a science: there are many useful tools, but implementation success ultimately depends on the capabilities of local staff to operate in complex systems.*<br><br>*ii) Develop local implementation staff with adaptive capabilities: complex systems are context-specific, inter-dependent, dynamically changing, and unpredictable.*<br><br>*iii) Bring people from different parts of the system together: if people can see value for themselves and stay engaged, they can work together to resolve inevitable conflicts between different parts of the system and find solutions.*<br><br>**B) Impact frameworks in action: Stephen Hanney (Brunel University London, UK) & Kathryn Graham (Alberta Innovates, Canada)**<br>*i) Impact assessment frameworks such as the payback framework are informed by models of collaboration and implementation.*<br><br>*ii) Application of impact assessment frameworks can help identify implementation pathways.*<br><br>*iii) Application of impact assessment frameworks for resource allocation provides incentives for and encourage research implementation.*<br><br>*iv) The Canadian Academy of Health Sciences impact framework: built on the Payback Framework's academic and societal impacts; provides a common set of tools, including indicators; identifies pathways to impact across the five payback categories; includes stakeholders needed to advance research to impact; has*

high face validity in Canada and elsewhere.

C) The view from within: learning from other research funders by asking 'how do you do it? Adam Kamenetzky (NIHR, UK)

*i) **Get set up:** funders need to consider upfront the resources required for embedded, relevant and methodical approaches to impact and its assessment.*

*ii) **Work together:** engaging researcher communities and wider stakeholders should be at the core of impact pathway planning, and subsequent research impact assessment.*

*iii) **Take time:** orienting research to societal and economic domains of impact is a long-term process of strategic change for funders and researchers.*

*iv) **Recognise benefits:** research impact assessment activities can lead to greater mutual understanding between funders and researchers, improved communications, and better evidence of value.*

D) ACTIONABLE INSIGHTS. Moderator: Pavel Ovseiko (University of Oxford, UK)

1: There are many frameworks and approaches that have already been or are still being tried and tested. Learning from others can help to learn about these frameworks' strengths and limitations, and how they play out in different contexts.

2: As more information on the use of the established frameworks and approaches is becoming available, and the development and implementations of new ones is continuing, it is becoming even more important for funders to commit sufficient resources to the assessment of research impact as well as to the implementation of research into practice. We should all learn from Steve Hanney on how to tirelessly and powerfully communicate this message, and not to shy away from doing this.

3: To support the growing impact ambitions of both funding and research organisations, greater efforts should be devoted to professionalising impact and implementation staff and developing their adaptive capabilities.

4: Engagement and communication are equally important, and we can learn from NIHR and Adam Kamenetzky personally how to effectively engage and communicate with stakeholders, including patients and the public, using innovative methods and approaches.

Session 2: Flipping the Switch – Cross-sector Learnings from Near and Far

Local, national and international panelists from sectors outside of health conveyed their experiences and perspectives on implementing scale and spread initiatives to achieve societal benefit.

A) Business innovation the Australian way: Peter Riddles (CSIRO, Australia)

i) The Australian economy is largely based on commodities (e.g. mining, agriculture) and services (e.g. education) - Innovation is often focused on the costs of production of those commodities, and is often acquired elsewhere and modified for purpose.

ii) More recently, government and industry are investing in creating more diversified economy based on various technologies - Innovation has a focus at the state (provincial) level and supported by federal government and national agencies (like CSIRO).

iii) Increasing emphasis on holistic approach to new industry development, examples include alternate energy (e.g. hydrogen); human bionics and medical technology; health services; space and aviation.

iv) Key drivers are sustainability for the environment, society and the economy.

B) Scale-and-spreading infrastructure nationally: Laura Hillier (CFI, Canada)

i) It is through strong collaboration linkages that new ideas become innovations and yield benefits to Canadians – research infrastructure can stimulate and support these collaborations.

ii) Scale and spread of innovation require momentum; this momentum can be built through investments in tools and people with a passion to solve important problems.

iii) Innovations and advances related to tool or technology development open new possibilities and can yield advances in other areas and solutions to new problems.

C) Clean technology the Alberta way: Elizabeth Shirt (ERA, Canada)

i) ***Toolset****: Technology and business model innovation – tools to identify and accelerate technologies (and companies) along the Technology Readiness Level (TRL) scale towards commercialization. Measurement and assessment – to determine if we are progressing on desired outcomes.*

ii) ***Team-set:*** *Collaboration – challenges are too big for one individual or one industry to tackle. Bringing together resources and removing barriers – Climate Change Innovation and Technology Framework (CCITF) and whole of Alberta approach as an example.*

iii) ***Mindset:*** *Evolving from linear to exponential way of thinking – big impact requires this shift. Look at challenges and see opportunity. Continuous improvement.*

D) ACTIONABLE INSIGHTS. Moderator: Dale Sanders (Health Catalyst, US)

1: Like an athlete who focuses their time on scoring at the neglect of physical conditioning, quite often, organizations strive for innovation but in so doing become distracted from performance on basic fundamentals. Master the fundamentals of your performance first, then make room on your calendar for innovation.

2: In order for change or innovation to achieve scalability and sustainability, it must appeal to the social norms, common sense, and behavioral economics of those people who are expected to change. Every investment strategy in innovation should include an evaluation of its likelihood of adoption based on the fundamentals of human behavior.

THEME TWO: CREATING A CULTURE FOR SOCIETAL IMPACT

Session 3: Building Stakeholder Engagement Platforms for Culture Change

Panelists reviewed stakeholder engagement approaches they have designed and implemented to affect culture change for optimizing use of R&I.

A) Creating an organizational culture to move implementation to impact: Reesa John (Alberta Innovates, Canada)

i) Need to consider that your project exists within a system that is made up of a community of people.

ii) Culture has the power to unite that community to ignite and inspire impact.

iii) Integrating a Culture plan up front as part of your Impact plan is critical to success.

B) Engaging stakeholders in the development of a system of Health Research Assessment (SARIS) in Catalonia: Núria Radó-Trilla (AQuAS, Spain)

When designing impact strategies it is important to:

i) Make the implicit changes explicit;

ii) Understanding the context, the stakeholders' challenges;

iii) Raise awareness of the need;

iv) Involving / engaging stakeholders (to be on the same page) - Need to give practical tools to stakeholders.

C) The importance of planning for effective adoption pathways to deliver social and economic impact: Tom Keenan (CSIRO, Australia)

i) Effective planning for impact is crucial for the ultimate delivery of that impact through the uptake and adoption of research 'babies'.

ii) Researchers must engage effectively with end users and other key stakeholders at appropriate stages across the impact pathway to ensure the desired impact is realized.

iii) Embedding impact culture in research organizations takes time – and requires both 'top down' and 'bottom up' approaches.

D) ACTIONABLE INSIGHTS. Moderator: Peter Riddles (CSIRO, Australia)

1: For successful and sustainable impact of scale and spread initiatives, cultural considerations, including the needs, interests, and values of the community of people engaged in the process, must be understood and built into impact plans upfront (prospectively). Culture is a core component of successful implementation and is present in all contexts.

2: Start by reflecting on the intentional outcomes you want to create. Ask the following: what kind of community and impact culture do you want to realize for the opportunity in question? What are the things the community needs to do, and what does the community value (in terms of behaviours and actions) that will help get you there and make the impact sustainable?

Session 4: Switching to Practice – Integrating Patients' Perspectives on What Matters

Panelists introduced real-world approaches for optimizing patients' contributions to inform what needs to be assessed in implementation and impact initiatives.

A) Meaningful participation of patients in R&I for implementation and impact: Jean Miller (Patient Representative (PaCER), Alberta, Canada)

i) First find out what's important to patients and study that;

ii) Then partner with patients throughout your research;

iii) Disseminate research results firstly and directly to patients: make patients the primary audience, not peer reviewed journals.

B) Importance of patient engagement with scale and spread of research and innovations into the health system: Jordan Antflick (Ontario Brain Institute, Canada)

i) The 'community' (service providers, charities, advocacy groups, grassroots organizations) plays an underappreciated role to support people living with brain disorders. People living with brain disorders receive most of their support outside the clinical setting.

ii) Co-developing tools and resources for patients empowers them with knowledge, levels the perceived power dynamic, and supports their ability to become active partners in their own treatment and care.

iii) Developing partnerships with community-based organizations extends the reach of these tools and helps scale and spread knowledge from the bottom up.

C) Engaging patients throughout the research lifecycle: Lauren Gerlach (AcademyHealth, US)

i) How might traditional definitions of evidence (including what's considered rigorous) need to change to

better incorporate patient narratives, insights from local communities, and other important data sources?

ii) What changes to the research enterprise (for example, in the areas of training and incentives) could help support meaningful engagement by patients in the production, dissemination, and implementation of research?

iii) How might we elevate and disseminate lessons learned about patient engagement in research to help advance this work moving forward?

D) ACTIONABLE INSIGHTS. Moderator: Tim Murphy (Alberta Innovates, Canada)
Insight 1: Patients are the primary audience for the results and efforts related to health research and innovation. A strong partnership between patients and researchers offers significant real benefits to both sides of the partnership. Partnership improves the quality and relevance of researchers' work and it also empowers the patient partners.

Action 1: Design and implement research and innovation programs and initiatives with this central partnership concept in mind.

Insight 2: Upstream inclusion of patients as partners in clinical and community settings – makes for more relevant, efficient and effective scale and spread of knowledge in the health ecosystem.

Action 2: Forging partnerships with community service providers (advocacy organizations, charities, etc.) extends the reach of knowledge and tools and assists in the scale and spread of knowledge – upfront and bottom up.

Insight 3: A re-engineering of the current evidence production process is required and will assist and support more effective production, dissemination, and implementation of research.

Action 3: Participants at this Summit are encouraged to identify, elevate, and disseminate use cases about patient engagement in research to help advance this field moving forward.

Session 5: Building a Diverse Talent Pool for the Future

Panelists spoke about innovative ways of diversifying and strengthening the talent pool of tomorrow to bridge the gap between research and innovation and impact.

A) Canadian health system impact fellowships: Meghan McMahon (CIHR-IHSPR, Canada)
i) PhD graduate employment trends are changing, learning health systems are emerging, and it's essential that doctoral/post-doctoral training keeps pace. The Health System Impact Fellowship program aims to address these trends and prepare PhD trainees and post-doc fellows for success and impact in a range of employment settings and roles within and beyond academia.

ii) Emerging lessons suggest five "program ingredients" are helping prepare fellows for stronger and more diverse careers: experiential learning within a health system organization, protected time for academic research, co-mentorship from health system and academic leaders, professional development training allowance, and participation in a national cohort.

iii) Health system organizations are keen to embed PhD talent as part of their teams, and PhD trainees and post-docs are keen for impact-oriented training opportunities: two years in, 62 health system organizations across the country have hosted 95 fellows.

B) Talent for the workforce of tomorrow: Alex Clark (University of Alberta, Canada)
i) Post-secondary institutions across Canada and internationally are increasingly supporting and rewarding diverse kinds of impacts more systematically. This means thinking and working beyond publications and

grants and creating cultures that support and incentivize impact.

ii) To promote internal capacity around impact - purchaser-based models which involve external 'expert' partners can bring expert input in from external partners.

iii) Policy and experts recommend that training and support be provided for impact across the career trajectory, including: to graduate students, post-doctoral fellows and researchers. Individuals enhancing their skill should engage in professional networks.

iv) 'Impact' skills need not be separate streams of professional development. Approaches integrating impact skills with other skills necessary for research success can provide added strategic value and help researchers develop impact skills more efficiently and strategically.

C) Gender equity pathway to impact: Pavel Ovseiko (University of Oxford, UK)
Great gender equity should:

i) Increase reproducibility of basic research;

ii) Enhance translation of clinical research;

iii) Maximize the potential of the scientific workforce.

***D)* Women's leadership and advancement in 20 Catalan biomedical research centers: Eduard Güell (AQuAS, Spain)**
i) There is an urgent need to introduce a gender perspective in health science.

ii) Research Impact Assessment (RIA) will be incomplete unless a gender perspective is introduced.

iii) Female leaderships is poor in science. Need to advocate for change.

iv) Assessment practices tend to fall into biases: our pilot in mental health helped us to see our own bias and the importance of including a gender perspective when assessing research impact.

E) ACTIONABLE INSIGHTS. Moderator: Adam Kamenetzky (NIHR, UK)
1: Equality, diversity and inclusion are fundamental aspects that ought to be considered at all levels of the research and innovation 'ecosystem': at an individual level, project level, program level, and the organizational/system level. Equity of impact cannot be achieved without efforts to understand and take action to address inherent and systemic biases: the work of AQuAS showed that while 'looking into the mirror' (to explore issues of gender bias in R&I) could be challenging, there was clear value in holding organizations to account, if collectively we are to move from being blind to these biases.

2: Evaluation thus has a key role to play – as in setting parameters for what will be evaluated, and how, we cannot lose sight of our values, and how these are reflected. Examples from CIHR on how embedded and reflective evaluation of their impact fellowship scheme, and its focus on developing 'non-traditional' competencies for those in hybrid/blended roles straddling academia, policy and practice, begin to show how these evaluations might reflect the values we ought to inspire and nurture, if we are to develop both a diverse and talented workforce.

Session 6: Concurrent Discussion Sessions

Session 6A): Connecting the Dots Between the Innovation Process and Societal Benefit: Moderator: Peter Riddles (CSIRO, Australia)

Description: Participants engaged in dialogue centred on unpacking the connection between the innovation process with economic and social development or a "systems" approach to innovation achieving impact.

i) *Innovation enablers include: shared vision, engagement, co-creation, problem solving, diversity of ideas, incentives, capacity development, space to think, collaborative spaces, safety to fail, manage workloads, data, resources, innovation grants, infrastructure, honest communications, culture, trust, champions, adaptability, persistence, common sense, continuous learning, understand naysayers, iteration, creating value, lean system, value-based system. Priorities for innovation can be identified by strengths, potential for impact, internal capabilities and gaps.*

ii) *System-wide influences on the system's/region's ability to capture broad and significant benefit from innovations include: rewards and copyright and other Intellectual Property (IP) instruments, but also shared ownership and the need to have people from diverse backgrounds at the table to develop multifaceted solutions given the mix of money, value, politics.*

iii) *To influence the system to perform better at 'capturing the benefit' the government needs to: achieve a balance of encouraging, enabling and enforcing, e.g. supporting culture changes and providing resources, but also holding people to account and being willing to shut down things that aren't delivering; be patient, both in terms of agreeing to broad end-goals then allowing researchers to adapt them to their context - not micromanaging; and working beyond short-term political cycles, including in holding people to account (closing the loop); and in practical terms, governments could provide infrastructure for engagement with wide range of stakeholders (e.g. to define benefits and their value), develop platforms for data sharing, use their own procurement practices to lead and establish markets, and help build public understanding and support for research.*

Session 6B): Addressing Sustainability in Real-world Applications: Moderators: Kelly Mrklas (AHS, Canada) and Rachel Flynn (University of Alberta, Canada)

Description: Participants engaged in focused conversation on navigating the quagmire of sustaining implementation efforts in the real-world.

i) *Sustainability requires the ongoing integration of stakeholders into existing structures/processes, and should consider both social norms and context. When new structures or processes are required, negotiated co-design among stakeholders is a vital and established expectation within the health care context. Co-develop a common language among stakeholders within an organization.*

ii) *Sustainability research needs the patient lens as a design-level lens, and needs to capture the constant evolution of change and quality improvement that occurs in health care and demonstrate how dynamic sustainability can be achieved (i.e. how to achieve stability in environments where evidence, contexts and other factors continually shift).*

iii) *A systems view may be helpful to understand sustainability. Role clarity and exploring one's own role from implementation to sustainability and impact can help focus efforts. The people part of change (e.g., change management, human factors) are a key consideration for understanding sustainability.*

iv) *Reported barriers to sustainability included barriers to sustainability monitoring/evaluation (e.g., lack of linked data, lack of standard metrics, challenges with ease of measurement, lack of feedback) and contextual barriers such as: resourcing (costs drive the decision making); implementation and learning climate (e.g., the complexity of health systems; silos within and across the healthcare system that prevent individuals from learning from one another around issues of common and mutual concern); and implementation readiness and culture (i.e., moving past the traditional "pilot project" mentality and shifting to longer lines of sight involving scale, spread, sustainability and impact).*

THEME THREE: NOVEL APPROACHES FOR SCALING RESEARCH AND INNOVATON TO IMPACT

Session 7: Accelerating Scale and Spread for Sustainable Impact – A Local Approach

Panelists reviewed innovative ways they have scaled-up and spread initiatives in the real-world, conveying practical approaches for sustaining impact.

A) Strategic considerations for research and innovation scale and spread: Tim Murphy (Alberta Innovates, Canada)

i) ***The Changing Landscape of Innovation:*** *From research dominant closed models of innovation – which features limited to no participation of subject matter experts; partners and suppliers/SME/MNE/ consumers and those with "lived" experience, etc. to open models of Innovation. Everyone in the open innovation dynamic can benefit, especially in healthcare by using both internal and external resources to accelerate innovation, organizations can capture value that would otherwise be lost to the system by facilitating ongoing engagement (1000 conversations).*

ii) ***The Changing Role of the Funder:*** *In the funding of Research and Innovation – from a "fund and forgetter" to Value Hunter/Value optimizer – funding models which allow investments in enabling platforms. To achieve true sustainable innovation/implementation along the Innovation Pipeline, a balanced portfolio is required, one that invests in the development of enabling capacity and infrastructure to accelerate innovations down the pathway, and one which features indicators and metrics which give line of sight to the intended impact, and we can measure it.*

iii) ***The Game-Changing impact of Emerging Technologies on the Innovation Pipeline****: Emerging technologies are reshaping healthcare in multiple ways including personalized and intuitive healthcare ecosystem – centred around the patients and their families into which their community of medical and social caregivers would be integrated. These technologies wrap around innovation in unique and cumulative ways, which accelerates the initiatives in the pipeline towards impact. The role of the funder and the funding models will need to permit the collisions to occur, to embrace new, open models of innovation.*

B) Co-creation approach for value - Partnership for Research and Innovation in the Health System (PRIHS): Nancy Fraser (Critical Care Strategic Clinical Network, AHS, Canada)

i) In the PRIHS all members are part of the network, coming together to solve problems and advance care. The collective wants to have an understanding of what is being generated in the research arena and have a bi-directional dialogue that allows the health system to identity gaps and feed them into the research community to help solve. We start to synthesize what is "known" that can help inform the problem/ gap we are trying to close.

ii) Then we 'test' what we have developed; funding sources serve as an enabling function to help move work down the pipeline shown in the Figure. What is learned at each stage of the pipeline circles around and informs the 'science'.

iii) Next, proven innovations are spread; on the pipeline figure there is an arrow so that sometimes things obviously ready for scale skip from stage 3 to 5. It is recognized that PRIHS doesn't guarantee an intervention entering a process of testing is ready for spread and scale at the end, however that would be the goal.

iv) Co-design of all work occurs across the pipeline with patients, families, researchers and operational providers involved. There are shifting focuses, with the research community "leading the dance" early on. As work moves to adoption and sustainability within the health system the clinical and operational leaders assume more of a lead to ensure the sustainability of the gains that have been discovered and are now being used.

C) Enhanced Recovery After Surgery (ERAS) - the Alberta Experience: Gregg Nelson (University of Calgary, Canada)

i) Enhanced Recovery After Surgery (ERAS) is a global surgical quality improvement program.

ii) ERAS was adopted in Alberta in 2013 with the hope of improving surgical care for patients.

iii) This started as a proof of concept project in colorectal surgery and since that time has evolved into a province-wide operationalized program now in multiple surgical areas and multiple hospitals.

D) Using implementation labs to clear a path to impact: Gabrielle Zimmermann (AbSPORU KT Platform, Canada)

i) One of the key initiatives of the KT Platform of the AbSPORU is to advance implementation science in Alberta. We do this in part by providing advice and assistance with the practical application of implementation science, but we are also exploring the development of an implementation science lab for long-term impact.

ii) Implementation Science (IS) labs are research teams embedded into the health care system to conduct studies using real world data to inform best practices.

iii) An IS lab can help build relationships and lay the foundation for robust processes within the healthcare system. The IS lab will provide an opportunity for systematic application of implementation science through process in a cohesive way, but will also contribute back to the science of implementation, for continual learning feedback.

E) Sustaining implementation efforts leveraging impact assessment: Kelly Mrklas (AHS, Canada) & Rachel Flynn (University of Alberta, Canada)

i) The importance of both the pipeline to orient change, investments in change, intent to scale-spread, sustain, and make impact, and of co-design in healthcare settings.

ii) Implementation and sustainability have much in common - recognize the importance of sustainability in the pipeline to impact, and the need for research to fill sustainability knowledge gaps.

iii) The research to fully describe, explain and demonstrate connections and to determine what is unique about implementation and sustainability in the pipeline, is innovative and emergent.

iv) SCNs are at the forefront of sustainability practice at-scale. Levering this innovation pipeline means applying co-design, implementation and sustainability rigor (case example: our SCN sustainability research study) to achieve impact.

F) ACTIONABLE INSIGHTS. Moderator: Jeffrey Crelinsten (Re$earch Money, Canada)

Insight 1: The pipeline is both a reflective tool and a line of sight that can be used to regularly embed the creation and use of high quality evidence for impact.

Action 1: Promote the use of the pipeline concept and provide training on its use to key stakeholders.

Insight 2: The goal of the healthcare system is to make patients' lives better. In this sense, patients are the "customer" and innovations must address their needs. However, governments are the payers, so there is often a disconnect between patient needs and government priorities.

Action 2: Actively engage patients, service providers and payers with researchers in the identification of gaps and problems and in the development and testing of solutions

Session 8: Show Me the Data

Panelists discussed data strategies and analytics that address data use in complex and dynamic

environments.

A) Data-driven insights for accelerating scale-up and spread: Dale Sanders (Health Catalyst, US)

i) Find the Truth, Tell the Truth, Face the Truth: The truth in today's US healthcare data is a fairly poor reflection of patient health. The data is a reflection of the healthcare delivery process and economic models, and less about actual patient health, treatment, and outcomes.

ii) Compulsory Quality Measures and Physician Burnout: By credible research, 65% of the compulsory measure in US healthcare are completely or most clinically invalid.

iii) Is Population Health Working in the US? Population health is about preventive medicine, but the US economic model, based on patient volume and fee-for-service medicine, is motivated by treatment, not prevention. What is working is very high touch, 24x7 availability to health services in the home, for patients who are 75+ years old, have 5+ chronic conditions, and are taking 8+ medications.

iv) Data Protectionism, Opacity, and Hoarding: We are hampered by poor data in US healthcare, but making poor data worse is the protection, hoarding, and reluctance to share and analyze what data we do possess. This is not about patient privacy. This is about personal, local policies and cultures that can be changed through leadership. This is about acknowledging that every piece of data that is collected as a consequence of healthcare delivery, is an artifact of data that ultimately belongs to the patient-- not healthcare systems, researchers, governments, or clinicians-- and should be shared and utilized to its fullest value for the benefit of patients. We are morally obligated to share, expose, and utilize patients' de-identified data to its fullest value, but we let our egos and insecurities stand in the way.

B) Semantic technologies and ontology-based data access for research impact monitoring and evaluation: Alba Velasco Trujillo (SIRIS Academics, Spain)

i) Evidence-based policy is an approach that appeals to data and evidence to introduce impactful changes by presenting carefully gathered evidence in favor of one solution rather than another. It is a way to explore scenarios and create arguments.

ii) Public policy and strategic decision making, in particular, suffer from lack of integration of existing data, which are available from separate and non-interoperable sources.

iii) Semantic Technologies solutions offer a disruptive toolkit to support the integration of data that are currently dispersed and highly heterogeneous and ensure they are accessed in an integrated, unified and semantically consistent way.

C) Navigating the Canadian data landscape: Rick Glazier (CIHR-IHSPR, Canada)

i) The data landscape in Canada includes several high functioning cross-jurisdictional data sources and platforms (e.g. Canadian Institute of Health Information (CIHI), Statistics Canada) but is mainly characterized by fragmentation and lack of comparability between and within sectors.

ii) Person-level community and social service encounters are not captured in any systematic way in most Canadian settings. Most health care encounters are captured digitally but siloed within sectors. In community settings they are locked within each provider's database and are largely inaccessible, even to the provider.

iii) Much work is needed to regulate, govern, set standards, remove access barriers, and harmonize digital health, community and social services data in Canada.

D) ACTIONABLE INSIGHTS. Moderator: Dale Sanders (Health Catalyst, US)

1: Every strategy for becoming "data driven" should dedicate a significant portion of the strategy and project plan to building trustful relationships with those affected by the data… both the providers of the

data and the consumers of the data.

2: Building trustful relationships around data is dependent on building a trustful, common semantic understanding of data. Every strategy for becoming "data-driven" should dedicate a significant portion of the strategy and project plan to achieving a common, semantic understanding of the data and metrics that underly the strategy.

Session 9: Engaged Assessment for Sustaining Impact

Panelists discussed ways of engaging stakeholders in measuring, assessing and improving impact at local, national, and international levels.

A) Social economic return on investment: Alan O'Connor (RTI, US) & Kathryn Graham (Alberta Innovates, Canada)

i) Having a shared vision of impact sustained the partnership over the years.

ii) Need a 'safe to test' and 'safe to fail' environment.

iii) New thinking, horizontal structures, integrated processes and practical tools were needed for decision making and implementation. Mixed methods and multiple data sources needed. Communicating impact requires the use of impact narratives.

iv) More engagement, feedback and co-design with the research community.

B) Using a co-creation and layered approach to scaling and spreading impact assessment in Canada: Maxi Miciak (Alberta Innovates, Canada)

i) Co-development and implementation of impact assessment frameworks and plans fuel stability through confidence, resonance, and relevance.

ii) Meeting users in the conditions that they live. Different groups are at different stages of readiness in terms of need, capacity, and ability, so providing options for engagement with impact assessment will promote sustainable scale and spread of impact assessment.

iii) Be patient AND be prepared for synergies when working with grass roots initiatives and stakeholders.

iv) Relationships and community are integration drivers, so provide opportunities for conversations and in-person meetings to develop.

C) Maximizing sustainable impact in The Netherlands: Wendy Reijmerink (ZonMw, Netherlands)

i) Don't talk about impact when the underlying evidence is not robust in terms of relevance and quality.

ii) Implementation science is all about adoption and adaption in real-world quality/innovation cycles.

iii) Smart funding agencies steer on accumulation of small wins, aimed at better health and healthcare for all.

D) Designing a monitoring, evaluation and learning approach to maximise sustainable impact for the UK National Institute for Health Research (NIHR)'s Global Health Portfolio: *Adam Kamenetzky & Carrie Hough (NIHR, UK)*

i) The UK National Institute for Health Research (NIHR) is developing a strategy for monitoring, evaluation and learning of its recently-established global health research funding portfolio, seeking to: collaboratively identify pathways to impact; establish mechanisms for testing whether these pathways do or do not work; identify emerging lessons to feed into portfolio development; and, embed monitoring evaluation and learning considerations throughout the portfolio;

ii) Activities to create an 'impact culture' for this work by engaging across the various points of the health research system have initially focussed on participatory activities with research applicants (via impact strengthening workshops to support applicants' conceptualization and planning of impact pathways) and peer review committee members (to establish their views on co-developing and testing an impact toolkit that can be scaled up outside of a workshop format, and the role of peer reviewers/committee members in critiquing applicants' impact plans). We have also co-developed a portfolio level Theory of Change to inform next steps;

iii) Key areas for further work include co-developing and testing relevance/suitability of a results framework for NIHR's global health portfolio (based upon the portfolio Theory of Change), and setting up proportionate monitoring and evaluation systems that support further capacity building for implementation and impact activities across the portfolio.

E) ACTIONABLE INSIGHTS. Moderator: Pavel Ovseiko (University of Oxford, UK)

1: Canadians in general and Albertans in particular have developed not only world-class, but in many respects world-leading expertise in implementation to impact. It builds on learnings from the world's best practice and is attuned to their unique context and needs. We need to tell the world about it as they themselves tend to be a bit too modest about their achievements.

2: Embedding impact into institutional policies and practices at ZonMW helps to advance implementation to impact activities but also creates a rich narrative around the institutional commitment to ensuring their sustainability. This narrative may well become a self-fulfilling prophesy and so worth considering and adopting in other organizations.

3: At NIHR, the sustainability agenda is reflected in its learning approach to a new Global Health Research program. It reminds me of “Push the Pace” approach at NIHR to continuously improve its processes. It may well be just a coincidence, but even so such a self-learning and reflective approach is also worth considering and adopting in other organizations.
